# Genome-Wide Identification and Characterization of Long Noncoding RNAs in *Populus × canescens* Roots Treated With Different Nitrogen Fertilizers

**DOI:** 10.3389/fpls.2022.890453

**Published:** 2022-05-12

**Authors:** Jing Zhou, Ling-Yu Yang, Xin Chen, Weng-Guang Shi, Shu-Rong Deng, Zhi-Bin Luo

**Affiliations:** State Key Laboratory of Tree Genetics and Breeding, Key Laboratory of Silviculture of the National Forestry and Grassland Administration, Research Institute of Forestry, Chinese Academy of Forestry, Beijing, China

**Keywords:** nitrate, ammonium, *Populus* × *canescens*, roots, lncRNAs, ceRNAs

## Abstract

Nitrate (NO_3_^−^) and ammonium (NH_4_^+^) are the primary forms of inorganic nitrogen acquired by plant roots. LncRNAs, as key regulators of gene expression, are a class of non-coding RNAs larger than 200 bp. However, knowledge about the regulatory role of lncRNAs in response to different nitrogen forms remains limited, particularly in woody plants. Here, we performed strand-specific RNA-sequencing of *P. × canescens* roots under three different nitrogen fertilization treatments. In total, 324 lncRNAs and 6,112 mRNAs were identified as showing significantly differential expression between the NO_3_^−^ and NH_4_NO_3_ treatments. Moreover, 333 lncRNAs and 6,007 mRNAs showed significantly differential expression between the NH_4_^+^ and NH_4_NO_3_ treatments. Further analysis suggested that these lncRNAs and mRNAs have different response mechanisms for different nitrogen forms. In addition, functional annotation of *cis* and *trans* target mRNAs of differentially expressed lncRNAs indicated that 60 lncRNAs corresponding to 49 differentially expressed *cis* and *trans* target mRNAs were involved in plant nitrogen metabolism and amino acid biosynthesis and metabolism. Furthermore, 42 lncRNAs were identified as putative precursors of 63 miRNAs, and 28 differentially expressed lncRNAs were potential endogenous target mimics targeted by 96 miRNAs. Moreover, ceRNA regulation networks were constructed. MSTRG.6097.1, MSTRG.13550.1, MSTRG.2693.1, and MSTRG.12899.1, as hub lncRNAs in the ceRNA networks, are potential candidate lncRNAs for studying the regulatory mechanism in poplar roots under different nitrogen fertilization treatments. The results provide a basis for obtaining insight into the molecular mechanisms of lncRNA responses to different nitrogen forms in woody plants.

## Introduction

Nitrogen is a vital nutrient for plants and has a strong influence on plant development (Oldroyd and Leyser, 2020). Plant roots play an important role in the acquisition and utilization of soil nitrogen (Wei et al., 2013; Jiao et al., 2018; Zhou et al., 2020). Plant roots mainly perceive nitrogen changes in the soil, activate the expression of key regulatory genes, such as small RNAs, transporter genes, and transcription factors, and then regulate the expression of genes related to root growth and development (Khan et al., 2011; Forde, 2014; Bellegarde et al., 2017; Massaro et al., 2019; Naulin et al., 2020; Oldroyd and Leyser, 2020). In this process, nitrate (NO_3_^−^) and ammonium (NH_4_^+^) not only serve as the main nutrients through which most plants acquire and utilize inorganic nitrogen but also play a crucial role in the plant response to nitrogen regulation as signaling molecules (O’Brien et al., 2016; Naulin et al., 2020; Wang et al., 2021). Although plants can use both ions, the physiological and molecular features of NO_3_^−^ and NH_4_^+^ are different for metabolism, which leads to distinct NO_3_^−^ or NH_4_^+^ preferences among plants (Patterson et al., 2010; Ruan et al., 2016). To sustain crop growth and development, obtaining a better understanding of the mechanisms of absorption and utilization of different nitrogen forms by crop roots is important.

Long non-coding RNAs (lncRNAs) are transcripts over 200 bp in length with no protein-coding capacity (Chekanova, 2015; Budak et al., 2020). LncRNAs play key regulatory roles in many important biological pathways by regulating the expression of *cis* and *trans* target mRNAs (Sun et al., 2018; Jha et al., 2020; Urquiaga et al., 2020). Moreover, lncRNAs can be used as precursors for miRNA biosynthesis (Wang et al., 2018) and as endogenous target mimics (eTMs) of miRNAs (Zhang et al., 2018). Therefore, there is a close regulatory relationship among lncRNAs, miRNAs, and mRNAs (Voshall et al., 2017). In recent years, studies on lncRNA-miRNA-mRNA regulatory networks have revealed that lncRNAs, as a type of competing endogenous RNA (ceRNA), competitively bind the same miRNA as mRNAs, which reduces the probability of miRNA-mRNA binding and increases the expression of mRNAs (Chen et al., 2021). To date, many lncRNAs involved in the nitrogen deficiency response have been identified in model plants (Chen et al., 2016; Fukuda et al., 2019, 2020; Liu et al., 2019; Lu et al., 2019; Wang et al., 2020a). For example, under low nitrogen conditions, 388 lncRNAs have been identified in poplars; among these, 126 lncRNAs responded to low nitrogen stress, 14 lncRNAs are predicted to be precursors of 25 miRNAs, and 4 lncRNAs are predicted to be target mRNAs of 29 miRNAs (Chen et al., 2016). Our preliminary research also showed that the lncRNA MSTRG.24415.1 is associated with the *cis* target mRNAs *TIP1.1* under low nitrogen stress and affects the wood formation process in poplar trees. According to the ceRNA theory, low nitrogen treatment results in downregulated expression of the lncRNA MSTRG.4094.1, which may promote the binding of mir5021-p5 to its target mRNA *TIP1.3* and thus lead to downregulated expression of *TIP1:3* (Lu et al., 2019). These studies suggest that lncRNAs participate in plant responses to nitrogen deficiency. In contrast, the identification and functional resolution of lncRNAs involved in plant responses to different nitrogen forms have not been reported.

Poplars, as model woody plants, are fast-growing trees that require a large amount of supplied nitrogen (Zhang et al., 2014). Different nitrogen fertilizations lead to contrasting morphological changes in poplar roots (Rewald et al., 2016) and to the regulation of distinct genes (Luo et al., 2015; Qu et al., 2016). Nevertheless, until now, how lncRNAs affect nitrogen uptake and the assimilation of different nitrogen forms in poplar to influence physiological and biochemical characteristics has not been studied. Therefore, investigating the regulatory mechanisms of lncRNAs in the absorption and assimilation capacity for different nitrogen forms in poplar roots is important.

In this study, *P.* × *canescens* saplings were exposed to 1 mM NaNO_3_, 500 μM NH_4_NO_3,_ and 1 mM NH_4_Cl for 21 days. This study aimed to perform an in-depth analysis of the regulatory mechanisms of lncRNAs in response to different nitrogen fertilization treatments in the roots of *P. × canescens*. To achieve this goal, we identified significant differential expression patterns of lncRNAs under treatment with different nitrogen forms, and the *cis* and *trans* target mRNAs of differentially expressed (DE)-lncRNAs were functionally annotated. Furthermore, DE-lncRNAs were identified as putative precursors of miRNAs and as potential endogenous target mimics. We also constructed lncRNA-miRNA-mRNA networks. The results provide new ideas for studying the regulatory mechanisms of woody plants in response to different nitrogen forms.

## Materials and Methods

### Plant Cultivation and Nitrogen Treatment

Seedlings of *P.* × *canescens* (*P. tremula* × *P. alba*, INRA 717-IB4 clone) plantlets were cultured in a tissue culture room for 4 weeks. The daily illumination time was 16 h, the photosynthetic photon flux density was 150 μmol m^−2^ s^−1^, the day/night temperature was 25/20°C, and the relative humidity was 50–55%. Subsequently, a set of 8 plants was planted in a hydroponic pot (10 pots in total) and irrigated with 8 l of Long Ashton (LA) nutrient solution every other day (Zhou et al., 2020). After 14 days in the greenhouse (under the same climatic conditions as the tissue culture chamber), plants with similar heights and ground diameters were selected, divided into 3 groups (twenty-two plants in each group), and transferred to a hydroponic system with nitrogen-free medium for 3 days (Balazadeh et al., 2014). Then, the three groups of plants were treated with different nitrogen fertilizers. The treatment conditions were as follows: one group of plants was treated with 1 mM NaNO_3_; the second group of plants was treated with the original LA nutrient solution as the control, which contained 500 μM NH_4_NO_3_; and the third group of plants was treated with 1 mM NH_4_Cl. The processing time was 21 days.

### Root Measurements and Harvesting

The plant root height and dry weight were measured before harvest. Eighteen plants were examined under three nitrogen fertilization treatments, and three biological replicates of each treatment were included in the experiment. Under the three nitrogen fertilization treatments, the dry weight of another four plants was calculated after the whole root of the plant was dried in a 60°C oven for 24 h. For harvesting, the whole root of each plant was dried with absorbent paper, wrapped in tinfoil, and immediately placed into liquid nitrogen. Each whole root sample was then ground into fine powder in liquid nitrogen with a ball mill (MM400, Retsch, Haan, Germany) and stored at −80°C.

To obtain sufficient test materials, the root organization of six plants that had been subjected to the same treatment was ground in equal amounts to obtain a mixed sample. As a result, 3 mixed samples of each treatment were obtained for further analysis. The NO_3_^−^ concentrations under the different nitrogen fertilization treatments were determined as described by Patterson et al. (2010), and the NH_4_^+^ concentrations under the different nitrogen fertilization treatments were analyzed spectrophotometrically according to the Berthelot reaction (Luo et al., 2013b).

### RNA Extraction and Sequencing

Total RNA was isolated from poplar roots using a total RNA extraction kit (TRK1001, LianChuan (LC) Science, Hangzhou, China). An RNA 6000 Nano LabChip Kit (5067–1,511, Agilent, CA, USA) and a Bioanalyzer 2,100 (Agilent, Santa Clara, CA, USA) were used to determine the quantity of total RNA. Total RNA from the NO_3_^−^, NH_4_NO_3,_ and NH_4_^+^ treatments was treated with RNase-free DNase I (E1091, Omega Bio-Tek, Norcross, GA, USA) to eliminate genomic DNA. For the sequencing of mRNAs and lncRNAs, a Ribo-Zero Gold Kit (MRZPL116, Illumina, CA, USA) was used to remove ribosomal RNA from the total RNA samples according to the kit instructions (Lu et al., 2019). Subsequently, cDNA libraries were established according to the protocol of the RNA-seq sample preparation kit (Illumina, CA, USA). Three cDNA libraries were constructed from each treatment level, and sequencing was performed according to the recommended protocol of the Illumina HiSeq 4,000 sequencer (Illumina, CA, USA) of LianChuan Science (Hangzhou, China). The raw sequence data were submitted to the Sequence Read Archive (SRA) under project ID PRJNA631840.

### Identification of lncRNAs and mRNAs

The raw sequence data were purified by removing low-quality (nucleotides with quality scores lower than 20, Q < 20), adaptor contamination and undetermined base reads, and then, FastQC (http://www.bioinformatics.babraham.ac.uk/projects/fastqc/) was used for quality verification to obtain clean reads. Using the TopHat2 package (version: 2.0.4), high-quality reads were blasted against the *P. tremula* × *P. alba* 717-1B4 genome v1.1 sequence (http://aspendb.uga.edu/index.php/databases/spta-717-genome), and up to two mismatches were allowed during the alignment process (Trapnell et al., 2009).

LncRNAs were identified according to the method described by Wang et al. (2017). In brief, the filter ratio was less than 50% of the coverage of the transcript, and transcripts with lengths less than 200 bp were removed. The coding potential of the remaining transcripts was then assessed using Coding Potential Calculator (CPC) software (Kong et al., 2007), and Coding-Non-Coding Index (CNCI) software (Sun et al., 2013) was used for assessment. Only transcripts with CPC scores less than −1 and CNCI scores less than 0 were considered lncRNA candidates.

The fragment per kilobase of exon per million fragments mapped (FPKM) algorithm was used to quantify the expression levels of lncRNAs and mRNAs as described by Pertea et al. (2015). Based on the FPKM value, the Ballgown package was used to calculate the differential expression levels of lncRNAs and mRNAs (Frazee et al., 2015). The log_2_(fold change) in DE-lncRNAs and DE-mRNAs was determined using the FPKMs of the genes under NO_3_^−^ or NH_4_^+^ treatments divided by those under the NH_4_NO_3_ treatments. The screening thresholds for identifying significantly DE-lncRNAs and DE-mRNAs were a *p-*value less than 0.05 and absolute values of log_2_(fold change) higher than 1.

### Target mRNA Prediction and Functional Analysis of DE-lncRNAs

To further explore the functions of DE-lncRNAs, potential *cis* and *trans* target mRNAs of the DE-lncRNAs were predicted (Zhang et al., 2018). The *cis* targets of DE-lncRNAs were predicted using a Python script designed by LianChuan Science (Lu et al., 2019). The target mRNAs in the 100-kb region upstream or downstream of the DE-lncRNAs were considered possible *cis* targets (Li et al., 2020). The *trans* targets of DE-lncRNAs were predicted based on the complementation effect of lncRNAs on the target mRNAs and RNA duplex energy (free energy less than −50) prediction using RIsearch. Then, GO (Gene Ontology) functional classification (Wang et al., 2017) and KEGG (Kyoto Encyclopedia of Genes and Genomes) pathway enrichment analysis (Masoudi-Nejad et al., 2007) were performed for the functional analysis of the differentially expressed *cis* and *trans* target mRNAs. Functional category analysis of the differentially expressed *cis* and *trans* target mRNAs was also performed using MapMan as described by Jia et al. (2017).

### Prediction of miRNA Precursors of lncRNAs

*P. × canescens* miRNA data were obtained using high-throughput sequencing, and 465 unique known miRNAs and 29 novel miRNAs were identified in the NO_3_^−^, NH_4_NO_3,_ and NH_4_^+^ libraries (Zhou and Wu, 2022a). The raw sequence data for the small RNAs were submitted to SRA under the project ID PRJNA631845. To predict which lncRNAs can be used as potential miRNA precursors, we compared the lncRNA sequences with mature *P. × canescens* miRNA sequences by BLAST and selected lncRNAs that showed an alignment with 100% homology to the mature miRNA sequence and the same chromosome as the mature miRNA.

### Construction of ceRNA Regulatory Networks

According to the method described by Sun et al. (2016), ceRNA regulatory networks among DE-lncRNAs, miRNAs, and DE-mRNAs were constructed. The miRNA data were based on the results of previous studies (Zhou and Wu, 2022a). The miRNA target mRNAs were predicted using Target Finder (mismatch score ≤ 2.5, penalty for mismatch and missing in strict matching zone is 1, G: U mismatch penalty is 0.5, and the penalty for non-strict matching zone is 0.5). Subsequently, target mimic prediction was performed to identify the complementary relationship between DE-lncRNAs and miRNAs. Using PsRobot, the sequences of DE-lncRNAs were entered into psRNA targets to identify miRNAs that may target DE-lncRNAs (mismatch score ≤ 2.5). Finally, the two complementary pairs were gathered to form the lncRNA-miRNA-mRNA regulatory network. The lncRNA-miRNA-mRNA regulatory network could be used as a ceRNA network if the following two conditions were met: (1) the expression patterns of interacting lncRNAs and mRNAs were up- or downregulated simultaneously because lncRNAs were reported to positively regulate the expression of mRNAs through lncRNA–miRNA–mRNA pairs (Ma et al., 2021); and (2) the interacting lncRNAs and mRNAs were significantly differentially expressed under different nitrogen fertilization treatments. The ceRNA regulatory network was imported into Cytoscape (v3.6.0) for visualization (version 3.6.0, http://chianti.ucsd.edu/cytoscape-3.6.0/).

### Real-Time Quantitative PCR Validation of the Significantly DE-lncRNAs and DE-mRNAs

To validate the expression of the ceRNA regulatory network, the DE-lncRNAs, and their target mRNAs, RT–qPCR validation was performed using SYBR Green assay reagents and a LightCycler^R^ 480 RealTime PCR System (Roche, USA) as described by Zhou et al. (2012). Total and small RNAs were extracted using the same samples used for RNA-sequencing. For small RNA reverse transcription, universal primers (Supplementary Table S1) in the Mir-X miRNA First-Strand Synthesis (Clontech Laboratories, CA, USA) were used for reverse transcription. Mature miRNA sequences and universal primers (Supplementary Table S1) were used for RT–qPCR according to the manufacturer’s instructions for the SYBR qRT-PCR kit (Clontech Laboratories, CA, USA). For RNA reverse transcription, 2 μg of total RNA was reverse-transcribed using the PrimeScript™ RT Reagent Kit (TaKaRa BIO, Japan). The specific primers of the tested genes are shown in Supplementary Table S1. Each DE-lncRNA, DE-miRNA, and DE-mRNA was analyzed in three replicates. Relative expression levels were calculated using the 2^–ΔΔCt^ method. *Actin* was used as the endogenous reference genes for DE-lncRNAs and DE-mRNAs, and *5.8S rRNA* was used as the endogenous reference gene for DE-miRNAs (Supplementary Table S1).

### Validation of lncRNA-miRNA-mRNA Pairs

To validate the ceRNA regulatory network, two lncRNA-miRNA-mRNA pairs were chosen, and transient coexpression analysis in *N. benthamiana* leaves was conducted as described by Zhou et al. (2020). Each of the eTMs (MSTRG.2693.1 and MSTRG.13550.1) and mRNAs (*PcNFYA2* and *PcCDL1*) were individually cloned into pCAMBIA1300 vectors under the control of the 35S promoter. Similarly, fragments of two miRNA precursors (mdm-miR169b_R-1 and miR171i-3p) were inserted into the pCAMBIA2300 vectors, which also carry a 35S promoter. Through electroporation, both vectors were then individually transformed into *A. tumefaciens* strain GV3101 and inoculated overnight at 28°C. Before infiltration into *N. benthamiana* leaves, an equal amount of *A. tumefaciens* cell culture containing the lncRNA and its corresponding miRNA and target mRNA was mixed as described by He et al. (2008). After 2 days of incubation in the dark, *N. benthamiana* leaves that were infiltrated were harvested for RT–qPCR. The gene-specific primers are shown in Supplementary Table S1. The tobacco *tubulin* gene was used as the endogenous reference gene of mRNAs (Supplementary Table S1).

### Statistical Analysis

Statgraphics software (STN, St Louis, MO, USA) was used for the statistical analyses of the data. Before statistical analyses of the data, the data were tested to determine the normality of their distribution. All the data were analyzed by one-way ANOVA using the different nitrogen treatment levels as a factor. The difference between the mean values was considered significant if the *p*-value from the ANOVA F test was less than 0.05.

## Results

### Phenotypic Responses of *P.* × *canescens* Roots to Different Nitrogen Forms

**Figure 1:**
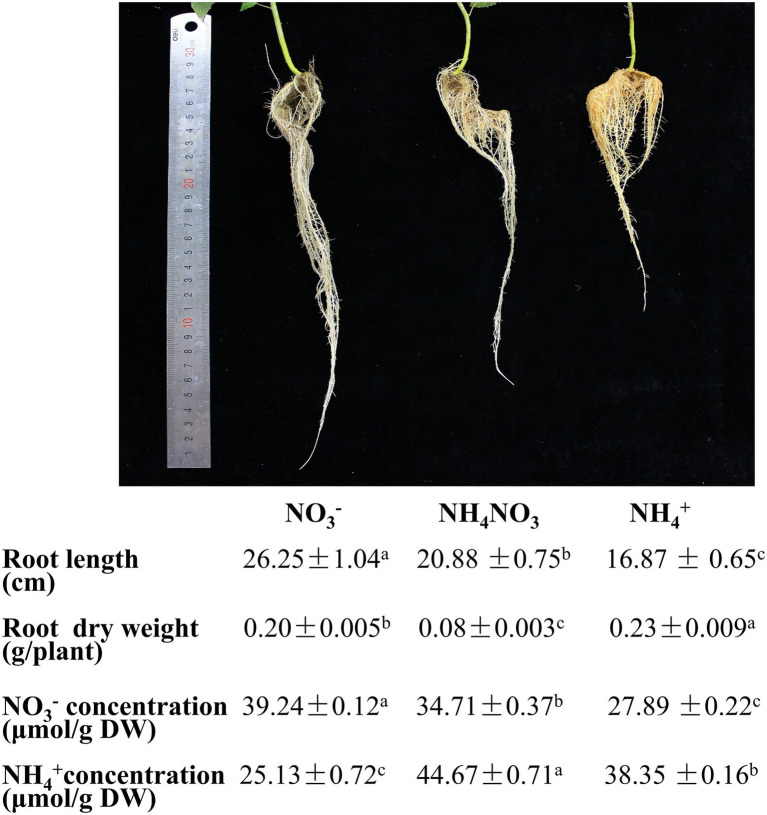
Morphological parameters and physiological indexes of *P. x canescens* roots under different nitrogen fertilization treatments for 21 days. Phenotypes of *P. x canescens* cultured under 1 mM NO_3_^-^, 500 μM NH_4_NO_3_ and 1 mM NH_4_^+^ for 21 days. Statistical analysis of root length, dry weight, NO_3_^-^ concentration and NH_4_^+^ concentration of roots. The data indicate the mean ± SE (n = 12). a, b, and c indicate significant differences based on ANOVA and Duncan's test *(P <* 0.05).

The influence of different nitrogen fertilization treatments on developing *P. × canescens* was monitored by measuring the root length and root dry weight. After long-term hydroponic cultivation (21 days), plants were supplied with NO_3_^−^, NH_4_^+^, or NH_4_NO_3_ (control). The plants growing under the NO_3_^−^ treatment exhibited longer roots and delayed growth of lateral roots compared with those growing under the NH_4_NO_3_ (control) treatment (Figure 1). The plants growing under the NH_4_^+^ treatment exhibited shorter roots and earlier growth of lateral roots than those growing under the NH_4_NO_3_ (control) treatment (Figure 1). Moreover, the root dry weight under the NO_3_^−^ and NH_4_^+^ treatments was higher than that under the NH_4_NO_3_ (control) treatment (Figure 1). However, there were no significant changes above plant height (Supplementary Figure S1). As different nitrogen forms may lead to different NO_3_^−^ or NH_4_^+^ concentrations in poplar roots, the concentrations in poplar roots were analyzed. The NO_3_^−^ treatment increased the NO_3_^−^ concentration by 13.05% and decreased the NH_4_^+^ concentration compared with that detected under the NH_4_NO_3_ (control) treatment. Moreover, the NH_4_^+^ treatment significantly reduced the NO_3_^−^ concentration by 19.65% and significantly increased the NH_4_^+^ concentration by 14.15% compared with those found under the NH_4_NO_3_ (control) treatment (Figure 1).

### Identification and Characterization of lncRNAs

cDNA libraries were constructed from *P. × canescens* root samples exposed to NO_3_^−^, NH_4_ NO_3,_ and NH_4_^+^ for 21 days and sequenced using an Illumina HiSeq™ 4,000 platform. Three biological repeats per treatment level were used to construct the libraries. High-throughput RNA-sequencing (RNA-seq) of these nine libraries led to the generation of 755, 579, 104 clean reads and 114.52 G clean bases (Supplementary Table S2). We subsequently mapped these clean reads to the *P. × canescens* reference genome to identify the transcripts (Zhou et al., 2015).

**Figure 2:**
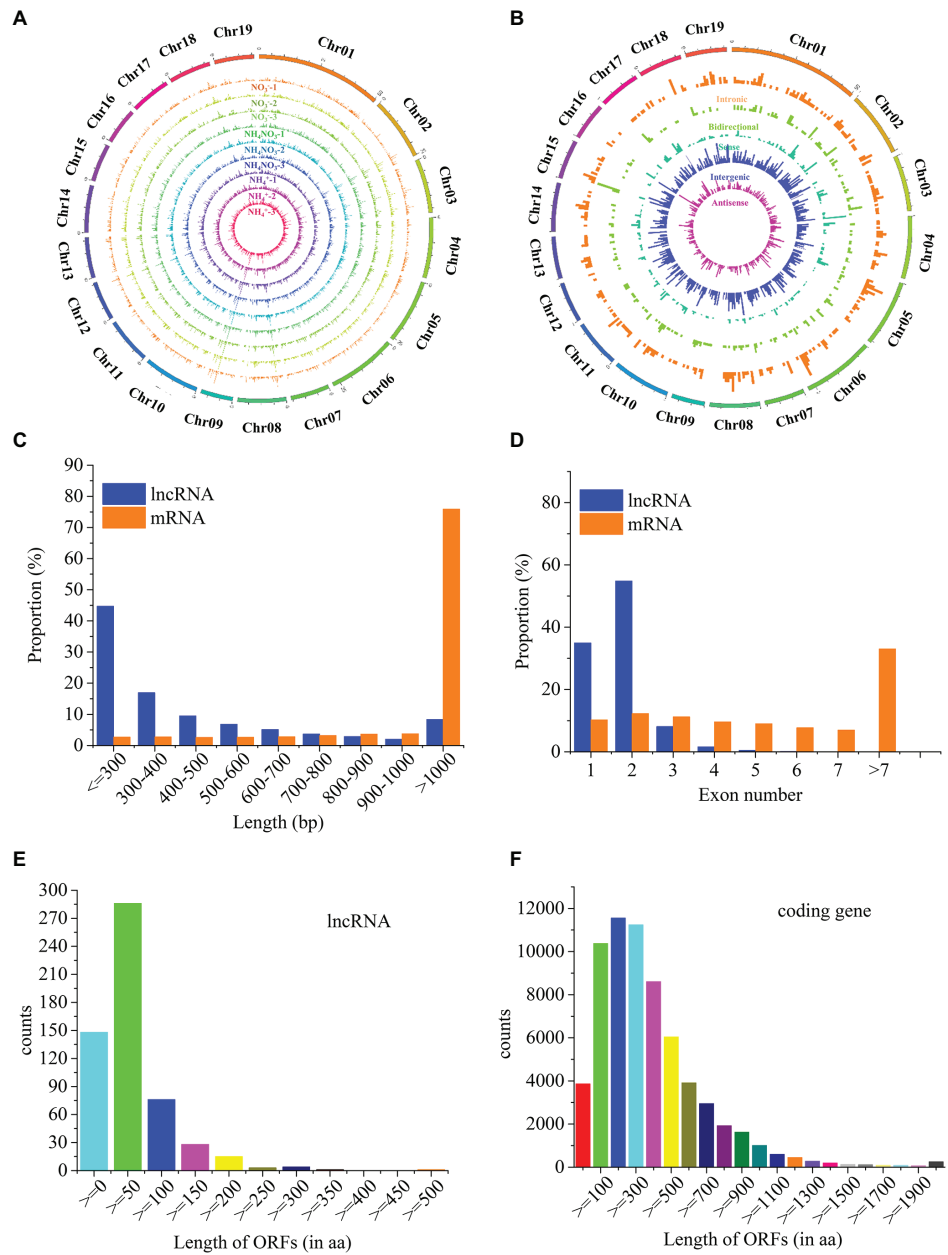
Comparison of structural features of lncRNAs and mRNAs. **(A)**, The expression level of lncRNAs along the 19 chromosomes. **(B)**, Chromosomal distribution of the five types of lncRNAs. **(C)**, Comparison of transcript lengths of lncRNAs and mRNAs. Blue represents lncRNAs, red represents mRNAs. **(D)**, Comparison of exon numbers of lncRNAs and mRNAs. Blue represents lncRNAs, red represents mRNAs. **(E)**, Open reading frame (ORF) length of lncRNAs. **(F)**, ORF length of mRNAs.

A total of 4,042 novel lncRNAs were identified in *P.* × *canescens* roots under the different nitrogen fertilization treatments (Supplementary Table S3). The 4,042 lncRNAs were evenly distributed across the chromosomes of poplar, without obvious location preferences (Figure 2A). These lncRNAs were divided into 1,500 intergenic, 1,191 antisense, 632 sense, 409 intronic, and 310 bidirectional lncRNAs based on their genomic locations (Figure 2B; Supplementary Table S3). The length, exon number, and open reading frame (ORF) length of 4,042 lncRNAs were compared with 73,013 transcripts from sequencing. The length of these lncRNAs was between 201 and 4,995 nt. In contrast, the length of approximately 45% of the identified lncRNAs was less than 300 nt, whereas 76% of the mRNAs were longer than 1,000 nt (Figure 2C). Approximately 90% of the lncRNAs were composed of one or two exons, whereas the exon number of mRNAs ranged from one to nine, and approximately 22% of the mRNAs contained seven or more exons (Figure 2D). Approximately 29% of lncRNAs did not have ORFs, and nearly 50% of lncRNAs contained short-chain (<50 residues) ORFs (Figure 2E); in contrast, 79.6% of mRNAs contained 100–700 ORFs (Figure 2F).

### Significantly Differentially Expressed lncRNAs Under Different Nitrogen Fertilization Treatments

**Figure 3:**
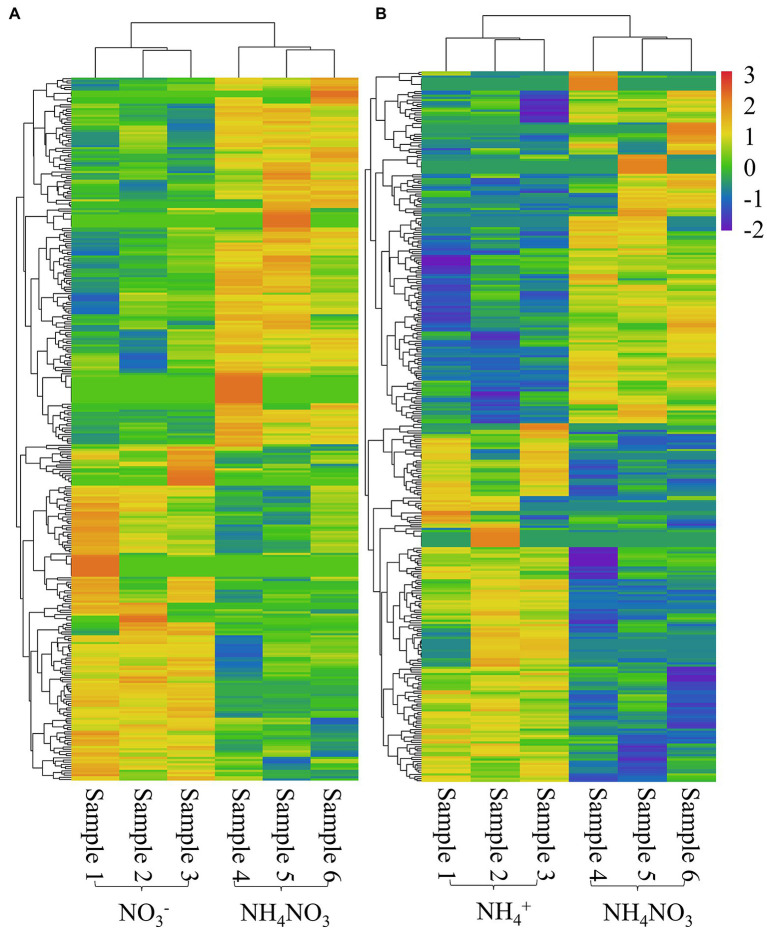
Heatmap of significantly differentially expressed lncRNAs with |log_2_(fold change)| > 1 and *P* value < 0.05 in the NO_3_^-^ vs. NH_4_NO_3_ **(A)** and NH_4_^+^ vs. NH_4_NO_3_ **(B)** comparisons of *P. x canescens.*

Among the 4,042 novel lncRNAs, 324 lncRNAs showed differential expression patterns between the NO_3_^−^ and NH_4_NO_3_ treatments, with a |log_2_(fold change)| value greater than 1 and a *p-value* less than 0.05, and 333 lncRNAs showed differential expression between the NH_4_^+^ and NH_4_NO_3_ treatments (Supplementary Table S4). The heatmaps of potential DE-lncRNAs are illustrated in Figure 3. Among the identified DE-lncRNAs between the NO_3_^−^ and NH_4_NO_3_ treatments, 154 lncRNAs were upregulated, and the remaining 170 were downregulated. Moreover, the analysis of the DE-lncRNAs between the NH_4_^+^ and NH_4_NO_3_ treatments revealed that 168 were upregulated, and the remaining 165 were downregulated (Supplementary Table S4). More interestingly, several DE-lncRNAs (MSTRG.5852.1, MSTRG.29402.1, MSTRG.22198.1, MSTRG.6743.1, MSTRG.24662.1, and MSTRG.5851.3) showed upregulated expression under the NO_3_^−^ treatment compared with the NH_4_NO_3_ treatments but downregulated expression under the NH_4_^+^ treatment compared with the NH_4_NO_3_ treatment. In addition, MSTRG.12063.1 expression was downregulated under the NO_3_^−^ treatment compared with the NH_4_NO_3_ treatments but upregulated under the NH_4_^+^ treatment compared with the NH_4_NO_3_ treatment. These results indicated that these lncRNAs have different mechanisms in response to different nitrogen forms in poplar roots. Eighteen DE-lncRNAs with a high number of reads were confirmed by RT–qPCR analysis (Supplementary Figure S2). Although the discrepancies in lncRNA expression levels did not match those obtained by sequencing in terms of magnitude, the trends of upregulation and downregulation were similar.

### Target Analysis and Functional Annotation of DE-lncRNAs

To globally identify mRNAs under *P. × canescens* exposure to NO_3_^−^, NH_4_NO_3_, and NH_4_^+^, a total of 73,013 mRNAs were identified from the nine libraries using high-throughput sequencing. Among the 73,013 mRNAs, 6,112 DE-mRNAs showing differential expression patterns, with |log_2_(fold change)| values higher than 1 and *p-values* less than 0.5, were found between NO_3_^−^ and NH_4_NO_3_. Moreover, 6,007 DE-mRNAs showed differential expression patterns between NH_4_^+^ and NH_4_NO_3_ (Supplementary Table S5).

To better analyze the roles of DE-lncRNAs, we analyzed the potential *cis* target mRNAs of DE-lncRNAs. Among these lncRNA-mRNA pairs, 276 differentially expressed *cis* target mRNAs were predicted between NO_3_^−^ and NH_4_NO_3_, and 265 differentially expressed *cis* target mRNAs were predicted between NH_4_^+^ and NH_4_NO_3_ (Supplementary Table S6). Moreover, *trans* targets of lncRNAs were also predicted. Among them, 561 potential *trans* target mRNAs, which were significantly differentially expressed, were predicted between NO_3_^−^ and NH_4_NO_3_, and 567 potential *trans* target mRNAs were predicted between NH_4_^+^ and NH_4_NO_3_ (Supplementary Table S7). In these networks, we found that the same lncRNA could be coexpressed with multiple transcripts, and multiple lncRNAs were coexpressed with one particular transcript.

**Figure 4:**
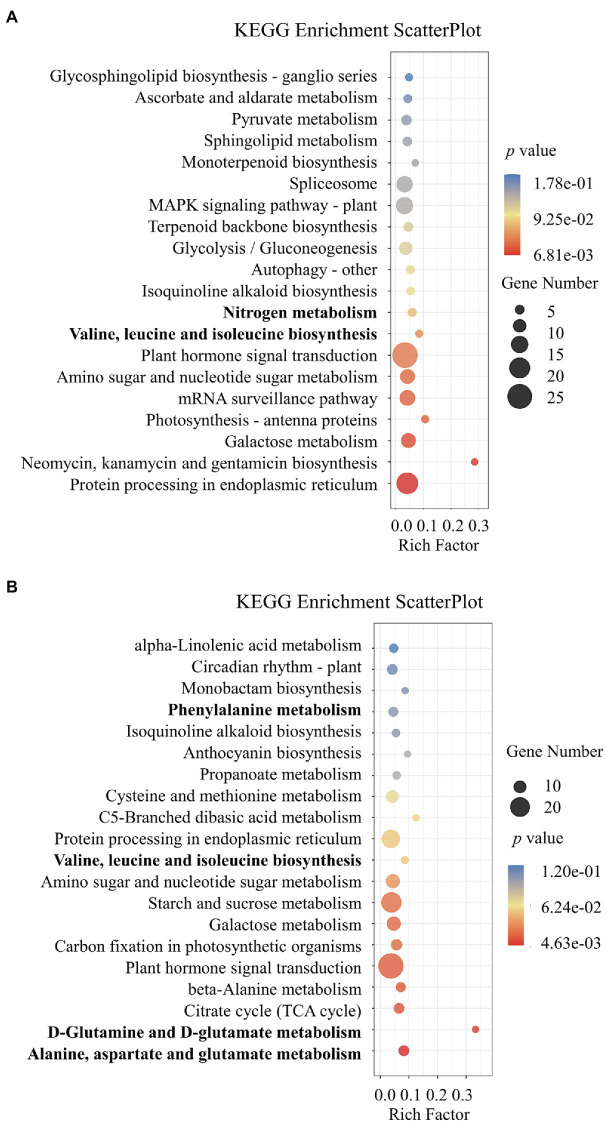
KEGG pathway analysis of identified differentially expressed *cis* and *trans* target genes. **(A)** represent NO_3_^-^ vs. NH_4_NO_3_ comparsion and **(B)** represent NH_4_^-^ vs. NH_4_NO_3_ comparsion.

To further understand the roles of lncRNAs of potential differentially expressed *cis* and *trans* target mRNAs, a KEGG analysis was performed to gain deeper insights into the functions of DE-lncRNA targets (Figure 4). KEGG analysis showed that the target genes obtained from the NO_3_^−^ vs. NH_4_NO_3_ comparison were involved in nitrogen metabolism and plant biosynthesis of amino acids, including valine, leucine, and isoleucine biosynthesis. The analysis of the target genes identified from the NH_4_^+^ vs. NH_4_NO_3_ comparison revealed that the biosynthesis of the amino acids valine, leucine, and isoleucine and metabolism of the amino acids alanine, aspartate, glutamate, D-glutamine, D-glutamate, cysteine, methionine, and phenylalanine were enriched pathways (Figure 4). These pathways are related to plant nitrogen physiological processes. A GO functional classification analysis was also conducted. The comparison of nitrogen forms at two different levels showed enriched GO terms (Supplementary Figure S3; Supplementary Tables S6, S7). Among the twenty-five biological processes, several main categories, including the regulation of transcription and response to stimulus processes, were enriched. Of the 15 cellular component categories, three important categories, namely, the nucleus, cytoplasm, and plasma membrane, were significantly enriched. Moreover, 15 molecular function categories were identified, and most of the target genes were enriched in the binding and enzymatic activity categories. This result indicated that *P. × canescens* DE-lncRNAs initiate broad and complex responsive processes that may play a role in binding and enzymatic activity-related functions to adapt to challenges imposed by different nitrogen form treatments (Supplementary Figure S3; Supplementary Tables S6, S7).

### lncRNA-mRNA Pairs Participate in Nitrogen Metabolism and Amino Acid Biosynthesis and Metabolism

To identify the lncRNA-mRNA pairs that play key roles in nitrogen metabolism and amino acid metabolism, functional categories were identified using MapMan. The functional categories of the *cis* and *trans* target genes of the DE-lncRNAs indicated that 60 DE-lncRNAs corresponding to 49 *cis* and *trans* target mRNAs were involved in plant nitrogen metabolism and amino acid biosynthesis and metabolism. In the NO_3_^−^ vs. NH_4_NO_3_ comparison, the MSTRG.2755.1-Potri.001G330900.3 (*sulfite oxidase*), MSTRG.30530.1-Potri.004G085400.2/3 (*glutamine synthetase cytosolic isozyme 1*), MSTRG.1921.1-Potri.004G085400.2/3 and MSTRG.7499.1-Potri.006G038400.4 (*ferredoxin-dependent glutamate synthase*) pairs participated in nitrogen metabolism. The MSTRG.2693.1-Potri.001G323100.1 (*protein AUXIN SIGNALING F-BOX 2-like*, *AFB2*) and MSTRG.20178.1-Potri.010G112800.6 (*PIN1-like auxin transport protein*) pairs participate in hormone metabolism and have been reported to participate in the NO_3_^−^ level response (Vidal et al., 2010). Thirty pairs also participated in amino acid metabolism. Four transporter peptides (*nitrate transmembrane transporter 1.5*; *NRT1.5*) and two transport-related amino acids (*amino acid transmembrane transporter 7*; *APP7*) also participated in the NO_3_^−^ level response (Table 1). In the NH_4_^+^ vs. NH_4_NO_3_ comparison, the MSTRG.28770.3-Potri.005G172400.1 (*nitrate reductase*), MSTRG.1771.1-Potri.007G047300.1 (*tRNA-dihydrouridine synthase*), MSTRG.441.1-Potri.012G113500.1 (*glutamate dehydrogenase 2 isoform X1*), MSTRG.17448.1-Potri.012G113500.1, MSTRG.1756.1-Potri.015G111000.1 (*glutamate dehydrogenase 2*) and MSTRG.17448.1-Potri.015G111000.1 pairs participated in nitrogen metabolism. Thirty-seven lncRNA-mRNA pairs participated in amino acid metabolism. Four transporter peptides (*NRT1.5*) and eighteen transport-related amino acids (*APP7*) also participated in the NH_4_^+^ level response (Table 1). To further analyze the relationship between DE-lncRNAs and their targets, which play key roles in nitrogen metabolism and amino acid metabolism, the expression patterns of the DE-lncRNAs and their targets under three nitrogen fertilization treatments were measured by RT–qPCR (Supplementary Figure S4).

**Table 1:** lncRNA-mRNA pairs involved in N-metabolism.

	LncRNA	mRNA	Function	cis location	trans energy	mRNA Annotation
NO_3_^−^ vs. NH_4_NO_3_	MSTRG.2755.1	Potri.001G330900.3	*cis*	100 K	NA	*sulfite oxidase*	
	MSTRG.2693.1	Potri.001G323100.1	*cis*	100 K	NA	*protein AUXIN SIGNALING F-BOX 2-like (AFB2)*	
	MSTRG.20178.1	Potri.010G112800.6	*cis*	100 K	NA	*PIN1-like auxin transport protein*	
	MSTRG.25619.1	Potri.001G084000.1	*trans*	NA	−51.42	*methionine gamma-lyase*	
	MSTRG.12407.3	Potri.001G185600.1	*trans*	NA	−52.43	*3-isopropylmalate dehydrogenase*	
	MSTRG.11893.1	Potri.001G267400.4	*trans*	NA	−52.67	*imidazole glycerol phosphate synthase hisHF, chloroplastic*	
	MSTRG.30521.1	Potri.003G088800.1	*trans*	NA	−51.18	*protein NRT1/PTR FAMILY 7.3 isoform X1*	
	MSTRG.30521.1	Potri.004G085400.2	*trans*	NA	−85.71	*glutamine synthetase cytosolic isozyme 1*	
	MSTRG.30521.1	Potri.004G085400.3	*trans*	NA	−85.71	*glutamine synthetase cytosolic isozyme 1*	
	MSTRG.1920.1	Potri.004G085400.2	*trans*	NA	−50.97	*glutamine synthetase cytosolic isozyme 1*	
	MSTRG.1920.1	Potri.004G085400.3	*trans*	NA	−50.97	*glutamine synthetase cytosolic isozyme 1*	
	MSTRG.28770.1	Potri.006G006000.1	*trans*	NA	−50.30	*oligopeptide transporter 3 isoform X1*	
	MSTRG.7499.1	Potri.006G038400.4	*trans*	NA	−51.52	*ferredoxin-dependent glutamate synthase, chloroplastic isoform X1*	
	MSTRG.25262.1	Potri.006G118400.2	*trans*	NA	−54.18	*threonine dehydratase biosynthetic, chloroplastic*	
	MSTRG.7791.2	Potri.006G118400.2	*trans*	NA	−50.21	*threonine dehydratase biosynthetic, chloroplastic*	
	MSTRG.24437.1	Potri.006G236000.1	*trans*	NA	−50.21	*amino acid permease 8*	
	MSTRG.24437.2	Potri.006G236000.1	*trans*	NA	−50.21	*amino acid permease 8*	
	MSTRG.1385.2	Potri.007G056900.1	*trans*	NA	−54.55	*oligopeptide transporter 7*	
	MSTRG.12407.2	Potri.007G056900.1	*trans*	NA	−51.31	*oligopeptide transporter 7*	
	MSTRG.28770.3	Potri.010G249600.3	*trans*	NA	−52.70	*D-3-phosphoglycerate dehydrogenase 2*	
	MSTRG.28770.1	Potri.010G249600.3	*trans*	NA	−52.70	*D-3-phosphoglycerate dehydrogenase 2*	
	MSTRG.28770.3	Potri.010G249600.1	*trans*	NA	−52.70	*D-3-phosphoglycerate dehydrogenase 2*	
	MSTRG.28770.1	Potri.010G249600.1	*trans*	NA	−52.70	*D-3-phosphoglycerate dehydrogenase 2*	
	MSTRG.28770.3	Potri.010G249600.2	*trans*	NA	−52.70	*D-3-phosphoglycerate dehydrogenase 2*	
	MSTRG.28770.1	Potri.010G249600.2	*trans*	NA	−52.70	*D-3-phosphoglycerate dehydrogenase 2*	
	MSTRG.4570.1	Potri.010G249600.3	*trans*	NA	−50.30	*D-3-phosphoglycerate dehydrogenase 2*	
	MSTRG.4570.1	Potri.010G249600.1	*trans*	NA	−50.30	*D-3-phosphoglycerate dehydrogenase 2*	
	MSTRG.4570.1	Potri.010G249600.2	*trans*	NA	−50.30	*D-3-phosphoglycerate dehydrogenase 2*	
	MSTRG.11893.1	Potri.010G249600.3	*trans*	NA	−50.23	*D-3-phosphoglycerate dehydrogenase 2*	
	MSTRG.11893.1	Potri.010G249600.1	*trans*	NA	−50.23	*D-3-phosphoglycerate dehydrogenase 2*	
	MSTRG.11893.1	Potri.010G249600.2	*trans*	NA	−50.23	*D-3-phosphoglycerate dehydrogenase 2*	
	MSTRG.28770.3	Potri.011G006800.2	*trans*	NA	−53.44	*mitochondrial glycine decarboxylase complex T-protein*	
	MSTRG.28770.1	Potri.011G006800.2	*trans*	NA	−53.44	*mitochondrial glycine decarboxylase complex T-protein*	
	MSTRG.28770.3	Potri.011G006800.1	*trans*	NA	−53.44	*mitochondrial glycine decarboxylase complex T-protein*	
	MSTRG.28770.1	Potri.011G006800.1	*trans*	NA	−53.44	*mitochondrial glycine decarboxylase complex T-protein*	
	MSTRG.12407.2	Potri.011G006800.2	*trans*	NA	−51.76	*mitochondrial glycine decarboxylase complex T-protein*	
	MSTRG.12407.3	Potri.011G006800.2	*trans*	NA	−51.76	*mitochondrial glycine decarboxylase complex T-protein*	
	MSTRG.12407.2	Potri.011G006800.1	*trans*	NA	−51.76	*mitochondrial glycine decarboxylase complex T-protein*	
	MSTRG.12407.3	Potri.011G006800.1	*trans*	NA	−51.76	*mitochondrial glycine decarboxylase complex T-protein*	
	MSTRG.16733.1	Potri.013G004100.1	*trans*	NA	−64.21	*S-adenosylmethionine synthase 2*	
	MSTRG.32721.1	Potri.013G004100.1	*trans*	NA	−51.73	*S-adenosylmethionine synthase 2*	
	MSTRG.24282.2	Potri.017G086500.4	*trans*	NA	−53.00	*cystathionine gamma-synthase 1*	
	MSTRG.12611.1	Potri.017G086500.4	*trans*	NA	−51.33	*cystathionine gamma-synthase 1*	
	MSTRG.31828.1	Potri.017G086500.4	*trans*	NA	−50.20	*cystathionine gamma-synthase 1*
NH_4_^+^ vs. NH_4_NO_3_	MSTRG.6004.1	potri.002 g236800.2	*cis*	100 K	NA	*aspartate kinase 3 (AK3)*	
	MSTRG.6005.1	potri.002 g236800.2	*cis*	100 K	NA	*aspartate kinase 3 (AK3)*	
	MSTRG.11374.1	Potri.005G162800.3	*cis*	10 K	NA	*3-deoxy-d-arabino-heptulosonate 7-phosphate synthase*	
	MSTRG.2374.1	Potri.001G283100.1	*cis*	10 K	NA	*3-chloroallyl aldehyde dehydrogenase (ALDH6B2)*	
	MSTRG.15812.1	potri.007 g137900.1	*cis*	100 K	NA	*aminotransferase*	
	MSTRG.24876.1	Potri.013G106300.1	*cis*	10 K	NA	*nitrate transmembrane transporter (NRT1.5)*	
	MSTRG.30693.1	Potri.017G152800.2	*cis*	10 K	NA	*nitrate transmembrane transporter (NRT1.5)*	
	MSTRG.22800.2	Potri.001G115600.1	*trans*	NA	−50.81	*amino acid binding (ACR3)*	
	MSTRG.11827.3	Potri.001G162800.1	*trans*	NA	−54.18	*alanine aminotransferase family protein*	
	MSTRG.29834.1	Potri.001G185600.1	*trans*	NA	−57.32	*3-isopropylmalate dehydrogenase*	
	MSTRG.11827.3	Potri.001G185600.1	*trans*	NA	−51.37	*3-isopropylmalate dehydrogenase*	
	MSTRG.7527.1	Potri.001G278400.1	*trans*	NA	−50.22	*asparagine synthetase [glutamine-hydrolyzing] 1 isoform X1*	
	MSTRG.11893.1	Potri.001G283100.1	*trans*	NA	−50.93	*methylmalonate-semialdehyde dehydrogenase*	
	MSTRG.10435.1	Potri.001G283100.1	*trans*	NA	−50.45	*methylmalonate-semialdehyde dehydrogenase*	
	MSTRG.16704.1	Potri.001G335300.4	*trans*	NA	−51.52	*lysine histidine transporter 1*	
	MSTRG.10531.1	Potri.001G348600.1	*trans*	NA	−58.46	*L-aspartate oxidase*	
	MSTRG.32480.2	Potri.001G348600.1	*trans*	NA	−50.37	*L-aspartate oxidase*	
	MSTRG.24329.1	Potri.004G013400.1	*trans*	NA	−52.01	*arogenate dehydratase/prephenate dehydratase 1*	
	MSTRG.24838.1	Potri.005G043400.1	*trans*	NA	−51.55	*bifunctional 3-dehydroquinate dehydratase/shikimate dehydrogenase*	
	MSTRG.28770.3	Potri.005G172400.1	*trans*	NA	−51.55	*nitrate reductase [NADH]*	
	MSTRG.28770.3	Potri.006G092000.2	*trans*	NA	−51.90	*protein NRT1/PTR FAMILY 5.6*	
	MSTRG.5625.1	Potri.006G123200.2	*trans*	NA	−73.66	*S-adenosylmethionine synthase 4*	
	MSTRG.17558.1	Potri.006G123200.2	*trans*	NA	−52.56	*S-adenosylmethionine synthase 4*	
	MSTRG.1771.1	Potri.007G047300.1	*trans*	NA	−51.41	*tRNA-dihydrouridine(16/17) synthase [NAD(P)(+)]-like*	
	MSTRG.18735.2	Potri.012G039000.7	*trans*	NA	−51.72	*glutamate decarboxylase*	
	MSTRG.441.1	Potri.012G113500.1	*trans*	NA	−50.31	*glutamate dehydrogenase 2 isoform X1*	
	MSTRG.17448.1	Potri.012G113500.1	*trans*	NA	−50.27	*glutamate dehydrogenase 2 isoform X1*	
	MSTRG.12407.2	Potri.013G049600.1	*trans*	NA	−55.82	*developmentally regulated GTP-binding protein*	
	MSTRG.11827.3	Potri.013G049600.1	*trans*	NA	−52.93	*developmentally regulated GTP-binding protein*	
	MSTRG.28770.3	Potri.013G049600.1	*trans*	NA	−51.10	*developmentally regulated GTP-binding protein*	
	MSTRG.12407.2	Potri.013G050200.1	*trans*	NA	−55.82	*developmentally regulated GTP-binding protein*	
	MSTRG.28770.3	Potri.013G050200.1	*trans*	NA	−51.10	*developmentally regulated GTP-binding protein*	
	MSTRG.28770.3	Potri.013G109500.1	*trans*	NA	−61.21	*serine acetyltransferase 5*	
	MSTRG.7527.1	Potri.013G109500.1	*trans*	NA	−54.91	*serine acetyltransferase 5*	
	MSTRG.12407.2	Potri.013G109500.1	*trans*	NA	−53.86	*serine acetyltransferase 5*	
	MSTRG.25742.1	Potri.013G109500.1	*trans*	NA	−52.65	*serine acetyltransferase 5*	
	MSTRG.11827.3	Potri.013G109500.1	*trans*	NA	−51.49	*serine acetyltransferase 5*	
	MSTRG.17448.1	Potri.015G111000.1	*trans*	NA	−52.07	*glutamate dehydrogenase 2*	
	MSTRG.1756.1	Potri.015G111000.1	*trans*	NA	−50.22	*glutamate dehydrogenase 2*	
	MSTRG.12057.1	Potri.016G113600.6	*trans*	NA	−53.70	*AUX1-like protein*	
	MSTRG.529.1	Potri.016G113600.6	*trans*	NA	−53.55	*AUX1-like protein*	
	MSTRG.1746.1	Potri.016G113600.6	*trans*	NA	−51.47	*AUX1-like protein*	
	MSTRG.16963.1	Potri.016G113600.6	*trans*	NA	−50.87	*AUX1-like protein*	
	MSTRG.24273.2	Potri.016G113600.6	*trans*	NA	−50.59	*AUX1-like protein*	
	MSTRG.17100.1	Potri.016G113600.6	*trans*	NA	−50.57	*AUX1-like protein*	
	MSTRG.8873.1	Potri.016G132200.2	*trans*	NA	−53.51	*alanine--glyoxylate aminotransferase 2 homolog 2*	
	MSTRG.11164.1	Potri.017G086500.1	*trans*	NA	−63.56	*cystathionine gamma-synthase 1*	
	MSTRG.17504.1	Potri.017G086500.1	*trans*	NA	−63.56	*cystathionine gamma-synthase 1*	
	MSTRG.11164.1	Potri.017G086500.3	*trans*	NA	−63.56	*cystathionine gamma-synthase 1*	
	MSTRG.17504.1	Potri.017G086500.3	*trans*	NA	−63.56	*cystathionine gamma-synthase 1*	
	MSTRG.11827.3	Potri.017G086500.1	*trans*	NA	−57.53	*cystathionine gamma-synthase 1*	
	MSTRG.11827.3	Potri.017G086500.3	*trans*	NA	−57.53	*cystathionine gamma-synthase 1*	
	MSTRG.1746.1	Potri.017G086500.1	*trans*	NA	−51.84	*cystathionine gamma-synthase 1*	
	MSTRG.1746.1	Potri.017G086500.3	*trans*	NA	−51.84	*cystathionine gamma-synthase 1*	
	MSTRG.12611.1	Potri.017G086500.1	*trans*	NA	−51.33	*cystathionine gamma-synthase 1*	
	MSTRG.12611.1	Potri.017G086500.3	*trans*	NA	−51.33	*cystathionine gamma-synthase 1*	
	MSTRG.31828.1	Potri.017G086500.1	*trans*	NA	−50.20	*cystathionine gamma-synthase 1*	
	MSTRG.31828.1	Potri.017G086500.3	*trans*	NA	−50.20	*cystathionine gamma-synthase 1*	
	MSTRG.28586.1	Potri.017G152800.2	*trans*	NA	−51.10	*protein NRT1/PTR FAMILY 5.8 isoform X2*	
	MSTRG.67.1	Potri.019G023600.1	*trans*	NA	−54.37	*developmentally regulated GTP-binding protein*	
	MSTRG.441.1	Potri.019G023600.1	*trans*	NA	−52.32	*developmentally regulated GTP-binding protein*	
	MSTRG.18735.2	Potri.019G023600.1	*trans*	NA	−51.89	*developmentally regulated GTP-binding protein*	
	MSTRG.31046.1	Potri.019G023600.1	*trans*	NA	−50.93	*developmentally regulated GTP-binding protein*	
	MSTRG.12407.2	Potri.019G023600.1	*trans*	NA	−50.88	*developmentally regulated GTP-binding protein*	
	MSTRG.28586.1	Potri.019G023600.1	*trans*	NA	−50.42	*developmentally regulated GTP-binding protein*

### LncRNAs as Precursors for Known and Novel miRNAs in *P.* × *canescens*

**Figure 5:**
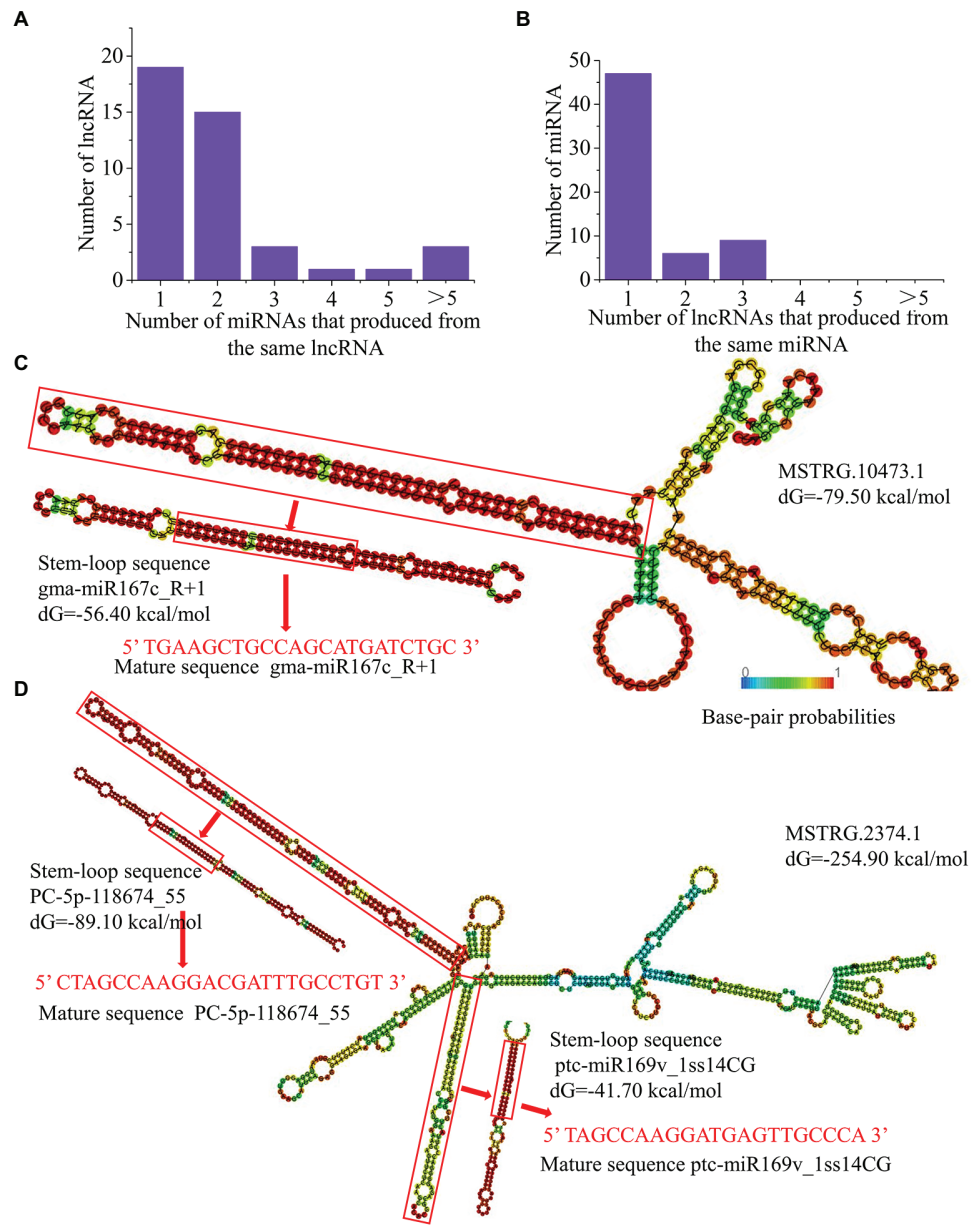
LncRNAs as precursors of miRNAs in *P. x canescens.* **(A)**, Number of miRNAs produced by lncRNAs. **(B)**, Number of lncRNAs that produce the same miRNA. **(C)**, Predicted secondary structure of MSTRG.10473.1, which contains one precursor sequence of gma-miR167c R+1. **(D)**, Predicted secondary structure of MSTRG.2374.1, which contains two precursors, namely, PC-Sp-118674_55 and ptcmiR169v lss14CG. The secondary structures were predicted using RNAfold. The color scale indicates high (red) to low (blue) probabilities of base pairing.

Previous studies have shown that lncRNAs can be associated with miRNAs as precursors of miRNAs (Wang et al., 2018). Using high-throughput sequencing, we identified 465 unique known miRNAs and 29 novel miRNAs in the NO_3_^−^, NH_4_NO_3,_ and NH_4_^+^ libraries (Zhou and Wu, 2022a). As a result, 42 lncRNAs were identified as precursors of 60 known miRNAs and 3 novel miRNAs in poplar roots (Supplementary Table S8). Among these, 23 lncRNAs can be used as precursors of two or more miRNAs, and the remaining 19 lncRNAs can only serve as precursors of one miRNA (Figure 5A). In addition, 47 miRNAs might be produced from one lncRNA, and the remaining 15 miRNAs might be generated by two or more different lncRNAs (Figure 5B). For example, MSTRG.12806.1/2 could be aligned with 6 miR396 family member precursors. Moreover, three lncRNAs (MSTRG.10473.1, MSTRG.4365.1, and MSTRG.17270.1) were aligned with 7 miR167 family member precursors in *P. × canescens.* In addition, one lncRNA (MSTRG.9926.1) matched well with precursors of two novel miRNAs (PC-3p-2543_1573 and PC-5p-360552_9; Supplementary Table S8). Therefore, an in-depth analysis of lncRNAs can provide a method for identifying novel miRNAs in plants.

To further verify whether lncRNAs have the typical stem–loop structures of miRNA precursors, the secondary structures of pre-miRNAs and lncRNAs were predicted with RNAfold. For example, the long arm of the lncRNA MSTRG.10473.1 corresponds to the stem–loop structure of the precursor of gma-miR167c_R + 1, which may be cleaved by an endonuclease complex to release the precursor sequence and ultimately form mature gma-miR167c_R + 1 (Figure 5C). The other lncRNA, MSTRG.2374.1, has two long arms, which was consistent with the stem–loop structure of the precursor sequences of the novel miRNAs PC-5p-118674_55 and ptc-miR169v_1ss14CG (Figure 5D). These lncRNAs can act as miRNA precursors to regulate the expression of mature miRNAs and are involved in nitrogen metabolism and amino acid biosynthesis and metabolism.

### LncRNA Transcripts as eTMs of miRNAs in *P.* × *canescens*

Recent studies have shown that lncRNAs can act as eTMs for miRNAs and work together to regulate mRNA expression in plants (Zhang et al., 2018). In our investigation, 14 eTMs were targeted by 38 miRNAs between NO_3_^−^ and NH_4_NO_3_, and 14 eTMs were targeted by 58 miRNAs between NH_4_^+^ and NH_4_NO_3_ (Supplementary Table S9). Among them, one eTM could be targeted by 1–3 miRNAs, whereas one miRNA could pair with several eTMs. For example, in the NO_3_^−^ vs. NH_4_NO_3_ comparison, MSTRG.6920.1 was targeted by 2 miRNAs belonging to the miR11607 and miR7486 family members. Specifically, MSTRG.2693.1 was targeted by a novel miRNA (PC-5p-118674_55) and miR169 family members (Supplementary Table S9). miR171 family members were identified in 3 eTMs (MSTRG.6097.1, MSTRG.27072.1 and MSTRG.13550.1). In the NH_4_^+^ vs. NH_4_NO_3_ comparison, miR396 family members were identified in 5 eTMs (MSTRG.12806.2, MSTRG.17795.3, MSTRG.33125.2, MSTRG.32317.2 and MSTRG.34633.2) (Supplementary Table S9). Specifically, MSTRG.3753.1 and MSTRG.8235.2 were targeted by 2 novel miRNAs, namely, PC-3p-256804_17 and PC-3p-134932_46, respectively (Supplementary Table S9).

### Construction of ceRNA Regulatory Networks

**Figure 6:**
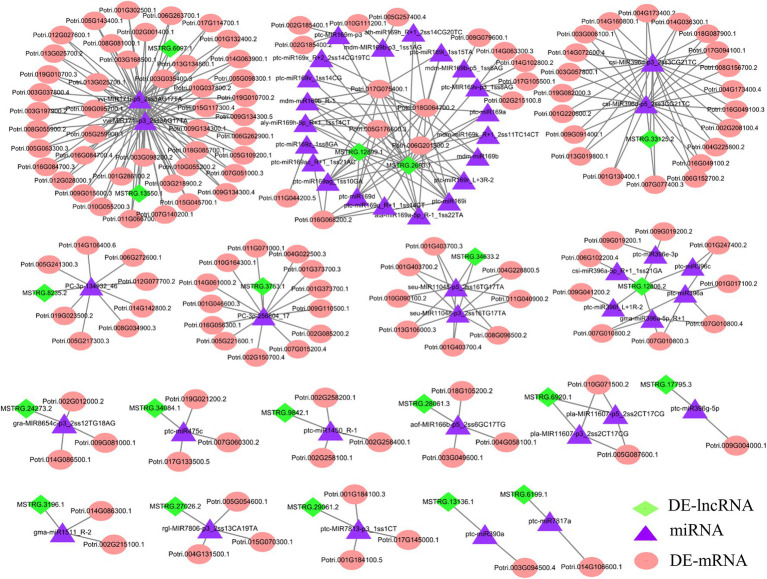
CeRNA networks in *P. x canescens* roots in response to different nitrogen forms. The green diamond-shaped, purple triangular, and orange round nodes represent DE-lncRNAs, miRNAs, and DE-mRNAs, respectively. Details regarding the abbreviations of the genes are listed in Supplementary Table S10.

**Figure 7:**
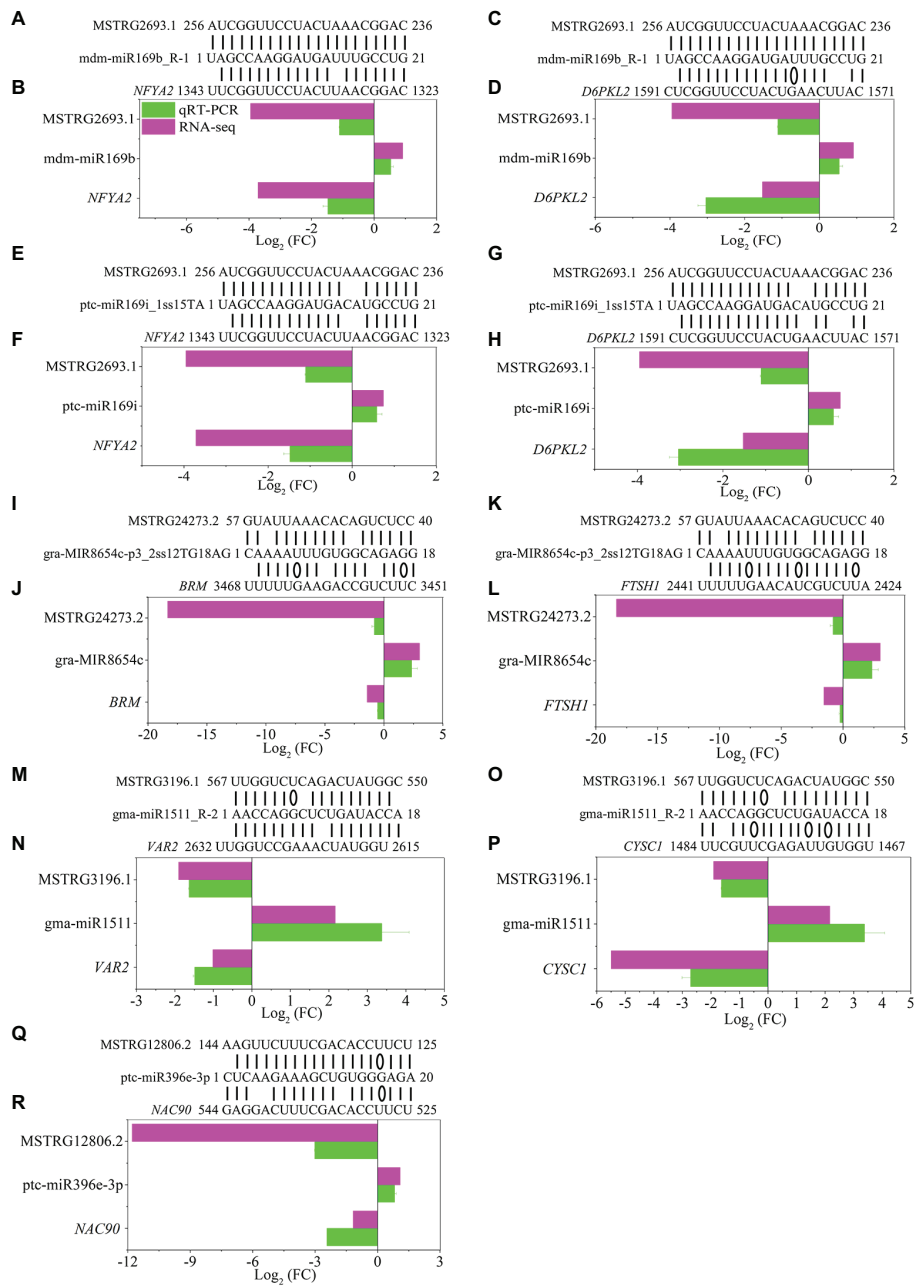
Validation of significantly differentially expressed lncRNAs, miRNAs and mRNAs under different nitrogen fertilization treatments in *P. x canescens* determined by sRNA-seq and RT—qPCR. **(B,D,F,H,J,I)** represent NO_3_^-^ vs. NH_4_NO_3_ comparsion and **(N,P,R)** represent NH_4_^+^ vs. NH_4_NO_3_ comparsion. The bars show the mean ± SE (n = 4). Sequence complementary positions of predicted lncRNA-miRNA-mRNA pairs **(A,C,E,G,I,K,M,O,Q)**. Details regarding the abbreviations of mRNAs are listed in Supplementary Table S10.

The development of regulatory network research has revealed that lncRNAs, as ceRNAs, participate in the regulation of target miRNA expression. Moreover, miRNAs can target mRNAs, inhibit target translation or degrade mRNAs (Qiu et al., 2012). To further explore the potential regulatory function of DE-lncRNAs, we examined the possible regulation of ceRNA networks in *P.* × *canescens* under different nitrogen fertilization treatments (Supplementary Table S10). The miRNA data were based on the results from previous studies (Zhou and Wu, 2022a). The network included 20 DE-lncRNAs, 47 miRNAs, and 143 DE-mRNAs (Figure 6; Supplementary Table S10). DE-mRNAs in the ceRNA regulatory network were assigned to functional categories using MapMan (Supplementary Table S10). Some functional categories, including amino acid metabolism and development, were associated with the regulation of nitrogen metabolism (Supplementary Table S10). Among the identified DE-mRNAs, *ATMS1* (*methionine synthase*), *CYSC1* (*CYSTEINE SYNTHASE C1*), and C2 domain-containing protein, which are targeted by MSTRG.8235.2, MSTRG.3196.1, and MSTRG.33125.2, are responsible for amino acid metabolism. Several targets, including the *NFYA1/2/6/7/11* (*nuclear transcription factor Y subunit A*), *ARF1/4* (*auxin response factors*), *MYB116 (myb domain protein 116),* and *NAC90* (*NAC transcription factor*) transcription factors, belong to the miR169, miR171, miR166, and miR396 families, respectively. These transcription factors may participate in nitrogen metabolism and plant growth and development (Supplementary Table S10). To further confirm the expression pattern of DE-lncRNAs and DE-mRNAs in ceRNA regulatory networks, we detected several lncRNA-miRNA-mRNA pairs under different nitrogen fertilization treatments by RT–qPCR and found that the DE-lncRNA expression patterns were consistent with those of their corresponding DE-mRNAs (Figure 7).

In the ceRNA network, we found that most nodes were connected to MSTRG.13550.1, MSTRG.6097.1, MSTRG.2693.1 and MSTRG.12899.1 (Figure 6; Supplementary Table S10). MSTRG.6097.1 was significantly upregulated in the NO_3_^−^ vs. NH_4_NO_3_ comparison and targeted two miR171 family members (vvi-MIR171i-p3_2ss3AG17TA and vvi-MIR171i-p5_2ss3AG17TA). Because MSTRG.6097.1 targets vvi-MIR171i-p3_2ss3AG17TA and vvi-MIR171i-p5_2ss3AG17TA as ceRNAs, 21 target mRNAs corresponding to two miR171 family members were significantly upregulated. Moreover, in the NH_4_^+^ vs. NH_4_NO_3_ comparison, MSTRG.13550.1 was significantly downregulated and targeted two miR171 family members (vvi-MIR171i-p3_2ss3AG17TA and vvi-MIR171i-p5_2ss3AG17TA), and 23 target mRNAs corresponding to two miR171 family members were also significantly downregulated. More interestingly, we found that among the 21 and 23 target mRNAs, two target mRNAs (Potri.013G025700.2 and Potri.013G025700.1), which encode *serine/threonine-protein kinase CDL1* (*CDL1*), coexisted under both treatments. However, the pattern of expression was indeed the opposite (Supplementary Table S10). Moreover, in the NO_3_^−^ vs. NH_4_NO_3_ comparison, 19 miR169 family members were targeted to the same lncRNA (MSTRG.2693.1), which was significantly downregulated, resulting in significantly downregulated expression of *NFYA3/10*. In the NH_4_^+^ vs. NH_4_NO_3_ comparison, 9 miR169 family members were targeted to the same lncRNA (MSTRG.12899.1), which was significantly upregulated, resulting in the significantly upregulated expression of *NFYA2* (Figure 6; Supplementary Table S10).

### Experimental Validation of Two lncRNA-miRNA-mRNA Pairs

**Figure 8:**
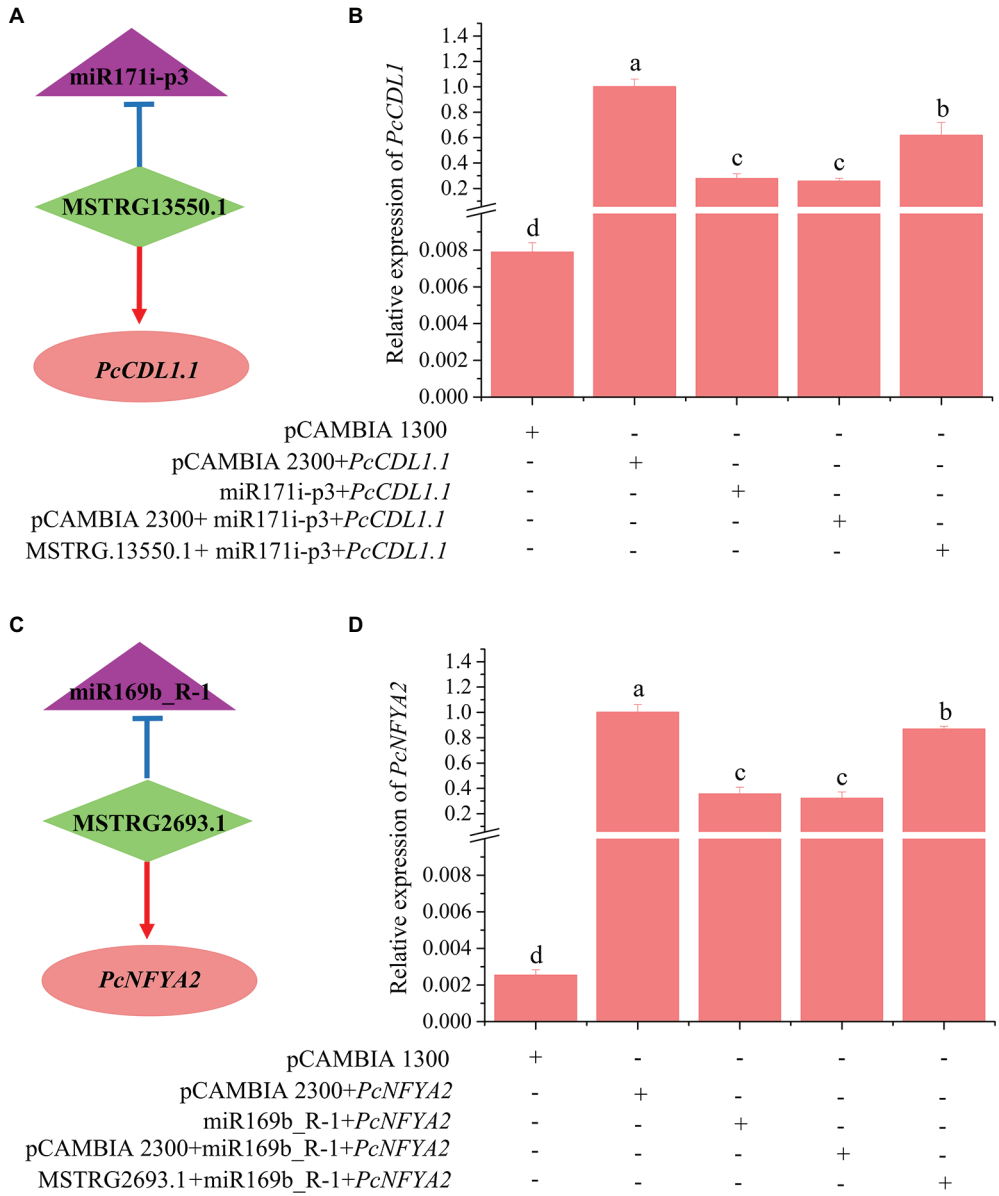
Validation of 1ncRNA-miRNA-mRNA pairs using *Nicotiana benthamiana* leaves transient coexpression system by RT—qPCR **(B,D)**. The bars show the mean ± SE (n = 4). Different letters on the error bars indicate significant differences. **(A)**, MSTRG.13550.1 targets miR171i-p3 and acts as an eTM for miR171i-p3; **(C)**, MSTRG2693.1 targets miR169b R-1 and acts as an eTM for miR169b R1.

To validate the role of lncRNAs as eTMs with miRNAs, we carried out transient coexpression in *Nicotiana benthamiana* leaves. Two lncRNA-miRNA-mRNA pairs were selected for the transient coexpression assays (MSTRG.13550.1-miR171i-*PcCDL1.1* and MSTRG.2693.1-miR169b-*PcNFYA2*). After 2 days of coexpression in *N. benthamiana* leaves, the upregulation of miR171i-p3 and miR169b_R-1 significantly decreased the transcript levels of the target mRNAs *PcCDL1.1* and *PcNFYA2*, respectively (Figure 8). Coexpression of miR171i-p3 with MSTRG.13550.1 restored the expression level of *PcCDL1.1* (Figures 8A,B). Similarly, MSTRG.2693.1 antagonized the inhibitory effects of mdm-miR169b_R-1 on the mRNA levels of *PcNFYA2* in poplars, and coexpression of mdm-miR169b_R-1 with MSTRG.2693.1 restored the expression level of *PcNFYA2* (Figures 8C,D). These results indicate that MSTRG.13550.1 and MSTRG.2693.1 likely antagonize the inhibitory effects of miR171i-p3 and mdm-miR169b_R-1 on the transcript levels of the target mRNAs *PcCDL1.1* and *PcNFYA2* in poplar roots, respectively.

## Discussion

### Poplar Roots Exhibit Adaptability and Plasticity Under Different Nitrogen Fertilization Treatments

Nitrogen is one of the essential mineral nutrients for plants and plays a very important role in regulating plant growth and development (Kung et al., 2013). NO_3_^−^ and NH_4_^+^, as the main inorganic nitrogen sources in soil, can be absorbed and utilized by plant roots (Oldroyd and Leyser, 2020). Studies have shown that different nitrogen fertilizations can alter the root morphological configuration of plants (Qu et al., 2016) and that the roles of NO_3_^−^ and NH_4_^+^ in root morphogenesis are different (Dai and Zhao, 2011; Luo et al., 2013a), which is due to the dynamic adaptation of the plant root morphological configuration to the dynamic adjustment of nitrogen forms supplied in soil (Hu et al., 2015). In *P. simonii × P. nigra*, after 21 days of treatment with different nitrogen fertilizations, the root length of poplar when NH_4_^+^ served as the only nitrogen source was lower than that obtained under the NO_3_^−^ and NH_4_NO_3_ treatments (Qu et al., 2016), and this finding is consistent with our results. In our study, after 21 days of being supplied with different nitrogen fertilizations, the roots under the NO_3_^−^ and NH_4_NO_3_ treatments were longer than those under the NH_4_^+^ treatment. Moreover, the root dry weight under NO_3_^−^ treatment was higher than that under NH_4_NO_3_ treatment, which was also consistent with the findings reported by Rewald et al. (2016). These results indicated that the root system of *P. × canescens* exhibited strong adaptability and plasticity to changes in the availability of different nitrogen forms.

### Many DE-lncRNAs in Poplar Roots Are Involved in the Response to Different Nitrogen Forms

In addition to physiological regulation, molecular regulation, particularly small RNA-mediated regulation, plays a key role in plant nitrogen absorption and assimilation, especially small RNA-mediated regulation (Raheel et al., 2018; Zhou et al., 2020; Zhou and Wu, 2022b). To date, research on small RNAs has focused on lncRNAs and miRNAs with regulatory functions (Qiao et al., 2018; Dos Santos et al., 2019; Lu et al., 2019). For example, in poplar wood, 91 DE-lncRNAs have been found under low nitrogen treatment. These lncRNAs participate in the regulation of wood properties and physiological processes of poplar under low nitrogen stress (Lu et al., 2019). However, the mechanism by which lncRNAs respond to different nitrogen forms in poplar roots has not been studied. In this study, genome-wide identification of lncRNAs was conducted, and a functional analysis of the DE-lncRNAs in poplar root responses to different nitrogen forms was performed. As a result, 324 and 333 lncRNAs showed differential expression patterns in the NO_3_^−^ vs. NH_4_NO_3_ and NH_4_^+^ vs. NH_4_NO_3_ comparisons, respectively. More interestingly, the same lncRNAs exhibited different expression patterns under different nitrogen fertilization treatments, which suggests that these lncRNAs have different response mechanisms for different nitrogen forms. More attention should thus be given to these lncRNAs, and their functions should be further studied.

### The Regulation of *cis* and *trans* Target mRNAs by lncRNAs Is Key for the Poplar Root Response to Different Nitrogen Forms

Previous studies have shown that lncRNAs can regulate the expression of their target mRNAs in both *cis* and *trans* manners depending on their neighboring gene and complementary pairing of bases (Li et al., 2020). To further resolve the biological functions of the DE-lncRNAs, the differentially expressed *cis* and *trans* target mRNAs of DE-lncRNAs were predicted, and the functions of these target genes were annotated. Both KEGG and MapMan analyses indicated that the *cis* and *trans* target genes of DE-lncRNAs play an important role in nitrogen metabolism, biosynthesis of amino acids, and plant amino acid metabolism. For example, MSTRG.2755.1 was downregulated in the roots of *P.* × *canescens* treated with NO_3_^−^, and the transcription of its potential *cis* target mRNA, Potri.001G330900.3, which encodes sulfite oxidase, was downregulated under NO_3_^−^ treatment (Supplementary Figure S4A). Potri.001G330900.3 is homologous to *Arabidopsis* sulfite oxidase (AT3G01910) and is reportedly involved in nitrate metabolism (Qiu et al., 2012). Potri.001G323100.1, a *cis* target mRNA of downregulated MSTRG.2693.1, was downregulated in the roots of NO_3_^−^-treated *P.* × *canescens* (Supplementary Figure S4B). This *cis* target mRNA is homologous to the *Arabidopsis protein AUXIN SIGNALING F-BOX 2-like* (*AFB2*). It has been reported that *AFB2* expression is induced by NO_3_^−^ treatment in *Arabidopsis* roots and affects their growth and development (Vidal et al., 2010). In addition, the lncRNAs MSTRG.6004.1 and MSTRG.6005.1 share the same *cis* target mRNA, potri.002 g236800.2, which is homologous to an *Arabidopsis aspartate kinase* (*AK3*) (AT3G02020) and is reportedly involved in amino acid biosynthesis and metabolism (Less and Galili, 2008; Jander and Joshi, 2009). Moreover, thirty and thirty-seven lncRNA-mRNA pairs identified from the NO_3_^−^ vs. NH_4_NO_3_ and NH_4_^+^ vs. NH_4_NO_3_ comparisons, respectively, participated in amino acid metabolism. Taken together, the results suggest that these lncRNA-mRNA pairs, as hub genes, might be essential for the response of *P.* × *canescens* roots to different nitrogen forms. Moreover, the expression of several hub lncRNA-mRNA pairs was verified by RT–qPCR (Supplementary Figure S4), which further confirmed the accuracy of the sequencing results.

### The Function of lncRNAs as miRNA Precursors and eTMs Is Crucial for the Poplar Root Response to Different Nitrogen Forms

Studies have shown that lncRNAs interact with miRNAs as miRNA precursors, target mimics, or targets to affect plant growth and development or in response to abiotic stress (Wu et al., 2013; Lu et al., 2019; Wang et al., 2020b; Feng et al., 2021). Interestingly, we found that the same lncRNA would correspond to multiple miRNA precursors or become an eTM of multiple miRNAs. Similarly, several lncRNAs may correspond to the same miRNA precursor or eTMs becoming the same miRNA (Wang et al., 2020b). This result demonstrated the existence of a complex regulatory interaction between lncRNAs and miRNAs. miR167 is the first reported miRNA involved in the nitrogen response (Gifford et al., 2008). In *Arabidopsis*, 5 mM NO_3_^−^ treatment for 12 h inhibits the expression of pericyclic cell miR167 and promotes the expression of its target *ARF8* (*AUXIN RESPONSE FACTOR 8*), which results in an effect on the root growth and development process (Gifford et al., 2008; Gutierrez, 2012). In this study, three lncRNAs (MSTRG.10473.1, MSTRG.4365.1, and MSTRG.17270.1) were aligned with 7 miR167 family member precursors involved in the nitrogen response in *P. × canescens* roots. This study further suggested that miR167 family members play a key role in regulating plant growth and development by participating in the response to different nitrogen forms. Moreover, this study provides a new idea for the regulatory mechanisms of miR167 family members involved in the response to different nitrogen forms and lays the foundation for studying the regulatory mechanisms of lncRNA-miR167 family members in response to different nitrogen forms.

### Competing Endogenous RNA Networks Are Crucial for the Poplar Root Response to Different Nitrogen Forms

Recent studies have shown that lncRNAs mainly interact with miRNAs as ceRNAs of miRNAs, which prevents the interaction between miRNAs and their target mRNAs and thereby enhances the function of encoding transcripts by inhibiting the translation of miRNAs to their target mRNAs (Qiu et al., 2012). The ceRNA network plays a key role in the response of plants to nitrogen deficiency (Chen et al., 2016; Borah et al., 2018; Shin et al., 2018; Lu et al., 2019). For example, in poplar trees, low nitrogen stress leads to downregulated expression of MSTRG.4094.1, which may promote the binding of mir5021-p5 to *TIP1.3* and thus lead to a reduction in *TIP1:3* transcription and a reduction in the vessel elements and lumina of fibers (Lu et al., 2019). However, the regulatory mechanism of ceRNA networks in poplar roots under different nitrogen forms has not been reported.

**Figure 9:**
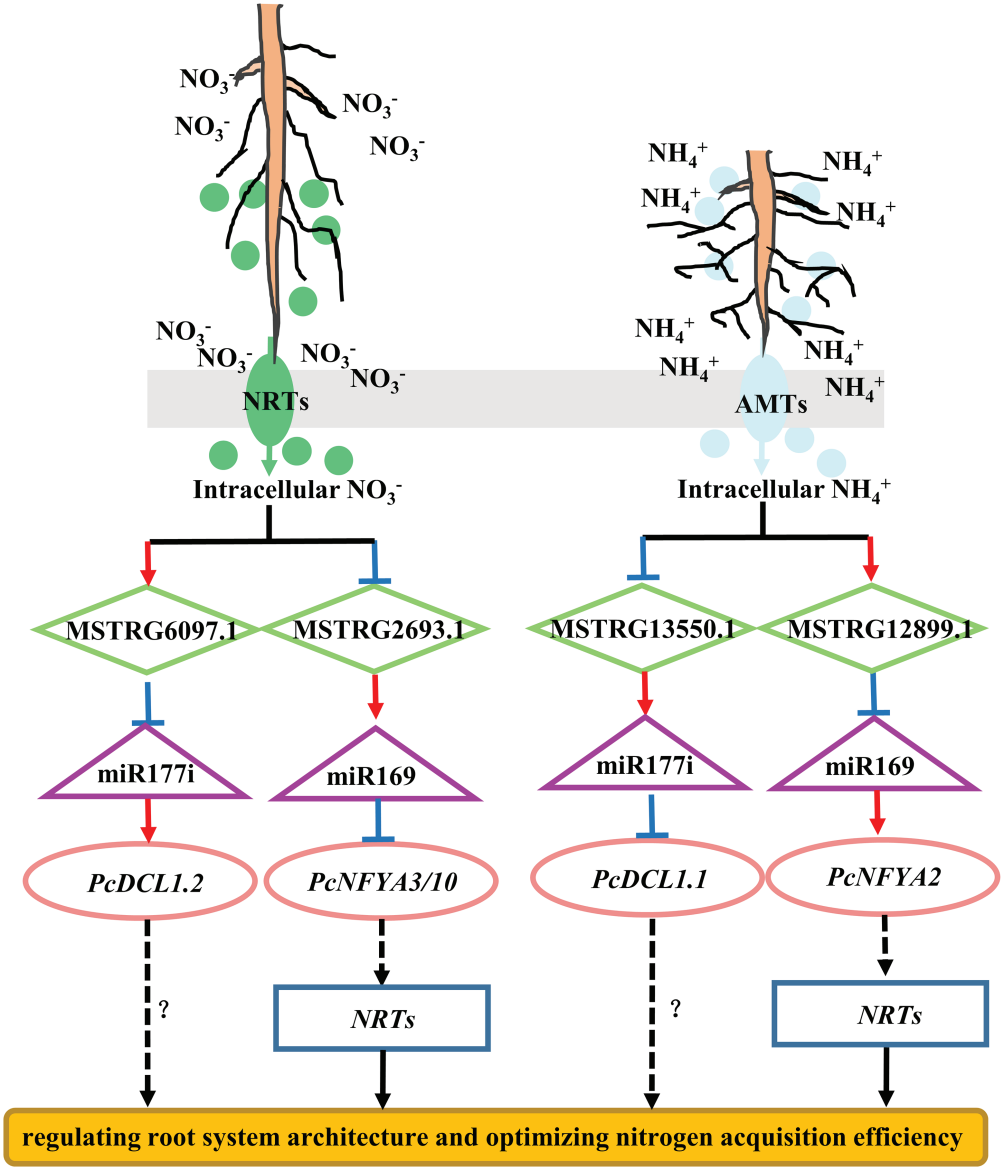
Simple model illustrating MSTRG.6097.1, MSTRG.13550.1, MSTRG.2693.1 and MSTRG.12899.1 as hub lncRNAs in the ceRNA networks. The target genes *CDL1* and *NFYAs* may enable woody plants to optimize nitrogen acquisition efficiency by regulating root system architecture and nitrogen uptake acticity under different nitrogen fertilization treatments. The arrow indicates positive regulation; the T-line represents negative regulation. The solid line represents the experimentally determined pathway, and the dotted line represents the predicted pathway.

In this study, the regulatory mechanism of ceRNA networks in poplar roots under different nitrogen fertilization treatments included 20 DE-lncRNAs, 47 miRNAs, and 143 DE-mRNAs (Figure 6; Supplementary Table S10). In the ceRNA regulation networks, most nodes were connected to MSTRG.6097.1 and MSTRG.13550.1. MSTRG.6097.1 and MSTRG.13550.1, as ceRNA targets for two miR171 family members, warrant attention. Two target mRNAs (Potri.013G025700.2 and Potri.013G025700.1), which encode *serine/threonine-protein kinase CDL1* (*CDL1*), coexisted in both treatment comparisons (NO_3_^−^ vs. NH_4_NO_3_ and NH_4_^+^ vs. NH_4_NO_3_), but their pattern of expression was indeed the opposite (Figure 9; Supplementary Table S10). The Potri.013G025700 gene is homologous to *Arabidopsis* serine/threonine-protein kinase (AT1G54820) and is reportedly involved in the nitrate response (Jander and Joshi, 2009).

Several studies have indicated that *NFYA* family members play a key role in the response to nitrogen by regulating *nitrate transporter* (*NRT*) gene family members in *A. thaliana* (Fukuda et al., 2019), wheat (Qu et al., 2015), rice (Yu et al., 2018) and poplar (Lu et al., 2019). In wheat, *TaNFYA*-*B1*, *TANRT1.1,* and *TANRT2.1* overexpression is induced, which increases NO_3_^−^ influx in wheat roots and promotes lateral root growth (Qu et al., 2015). In previous studies, we found that the *NFYA* transcription factor, as the target mRNA of miR169 family members, participates in the alteration of poplar root morphology in response to different nitrogen forms (Zhou and Wu, 2022a). The network obtained in this study also identified several members of the miR169 family (Figure 6; Supplementary Table S10). In the NO_3_^−^ vs. NH_4_NO_3_ comparison, 19 miR169 family members were targeted to the same lncRNA (MSTRG.2693.1), resulting in significantly downregulated expression of *NFYA3/10*. In the NH_4_^+^ vs. NH_4_NO_3_ comparison, nine miR169 family members were targeted to the same lncRNA (MSTRG.12899.1), resulting in significantly upregulated expression of *NFYA2*. These results suggest that different lncRNAs can target different members of the same miRNA family and simultaneously act on different targets under different nitrogen fertilization treatments. Moreover, different members of the same gene family responded to different nitrogen form treatments, which further indicates that the response mechanisms of woody plant roots to different nitrogen forms are different. Therefore, a complex mechanism may exist for ceRNA regulation networks to regulate the expression profile of lncRNAs, miRNAs, and their targets.

In conclusion, MSTRG.6097.1, MSTRG.13550.1, MSTRG. 2693.1, and MSTRG.12899.1, as hub lncRNAs in ceRNA regulation networks, are potential candidate genes for studying the regulatory mechanism in poplar roots under different nitrogen form treatments. Moreover, in the ceRNA network formed by the four candidate hub lncRNAs, the target mRNAs *CDL1.1/2* and *NFYAs* may enable woody plants to optimize nitrogen acquisition efficiency by regulating the root system architecture and nitrogen uptake activity under different nitrogen form treatments (Figure 9). Verification of this hypothesis will require further functional analysis of these candidate lncRNAs through experimental investigation. The results of this study provide clues to comprehensively elucidate the physiological and molecular mechanisms of poplar root responses to different nitrogen forms.

## Data Availability Statement

The data presented in the study are deposited in the NCBI repository, accession numbers PRJNA631840, https://dataview.ncbi.nlm.nih.gov/object/PRJNA631840 and PRJNA631845, https://dataview.ncbi.nlm.nih.gov/object/PRJNA631845.

## Author Contributions

JZ conceived the experiment and performed most of the experimental work. JZ, L-YY, and XC performed the experiments and data analyses. JZ, W-GS, S-RD, and Z-BL interpreted the experimental data and wrote the manuscript. All authors contributed to the article and approved the submitted version.

## Funding

This work was supported by the National Natural Science Foundation of China (grant nos. 32171739 and 31500507) and the Fundamental Research Funds for the Central Nonprofit Research Institution of CAF (grant no. CAFYBB2016QB005).

## Conflict of Interest

The authors declare that the research was conducted in the absence of any commercial or financial relationships that could be construed as a potential conflict of interest.

## Publisher’s Note

All claims expressed in this article are solely those of the authors and do not necessarily represent those of their affiliated organizations, or those of the publisher, the editors and the reviewers. Any product that may be evaluated in this article, or claim that may be made by its manufacturer, is not guaranteed or endorsed by the publisher.
