# Supplementary figures and images for: Genome-Wide Identification and Characterization of Long Noncoding RNAs in Populus × canescens Roots Treated With Different Nitrogen Fertilizers
Source: Front Plant Sci. 2022 May 12;13:890453. doi: 10.3389/fpls.2022.890453 (PMC9135444; doi:10.3389/fpls.2022.890453)

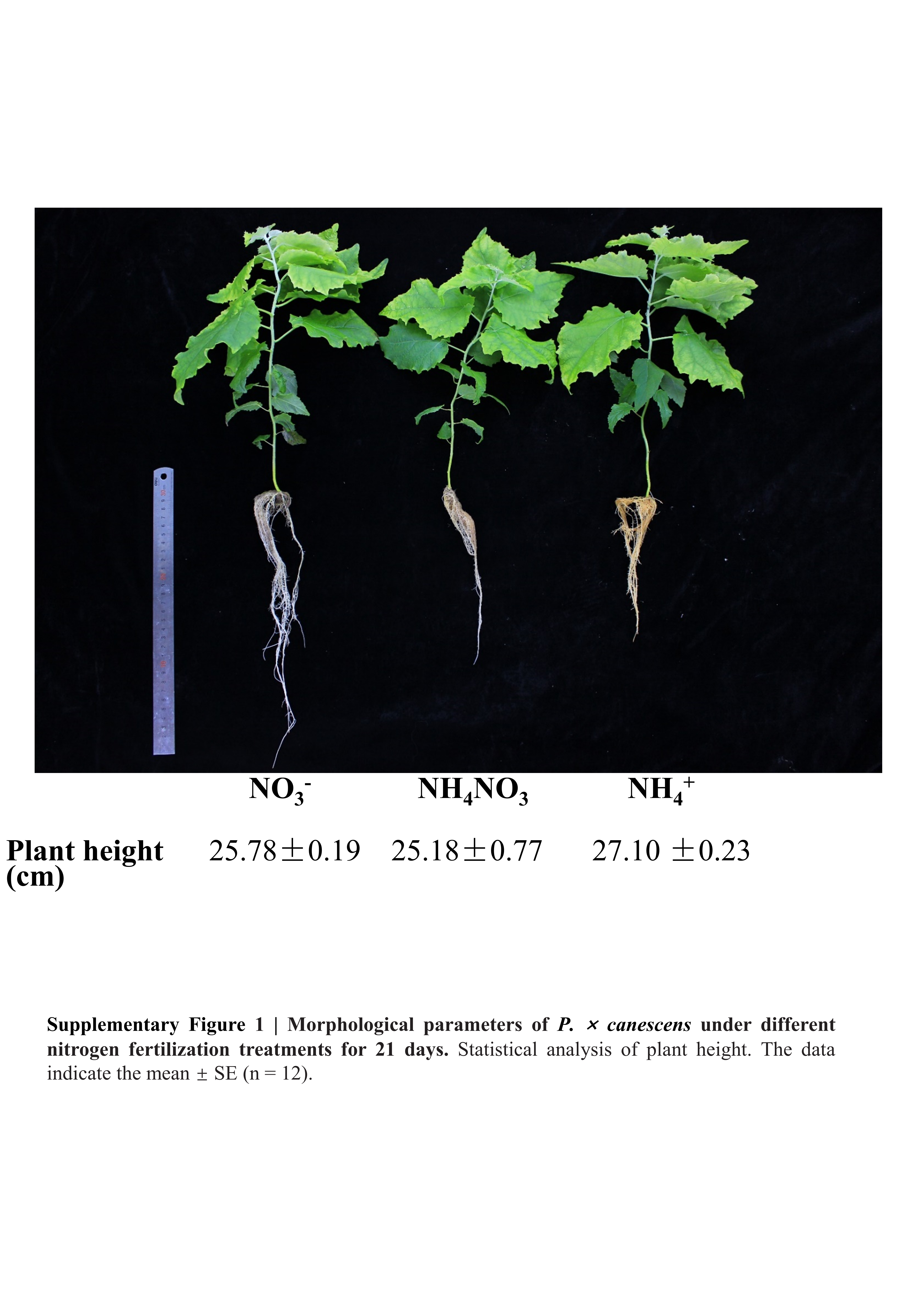

Supplement: Supplementary file 2 [file Image_1.TIF]

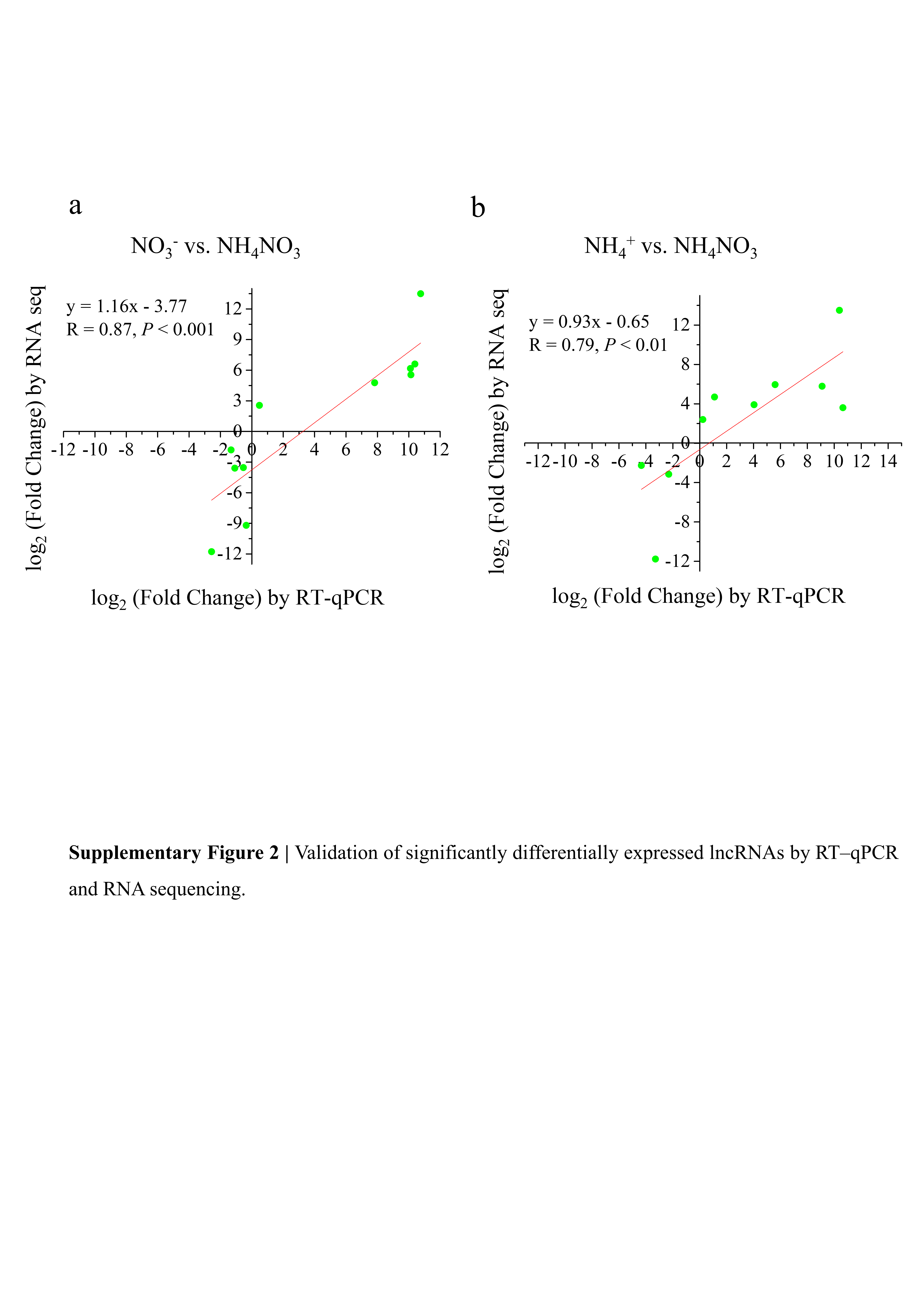

Supplement: Supplementary file 3 [file Image_2.TIF]

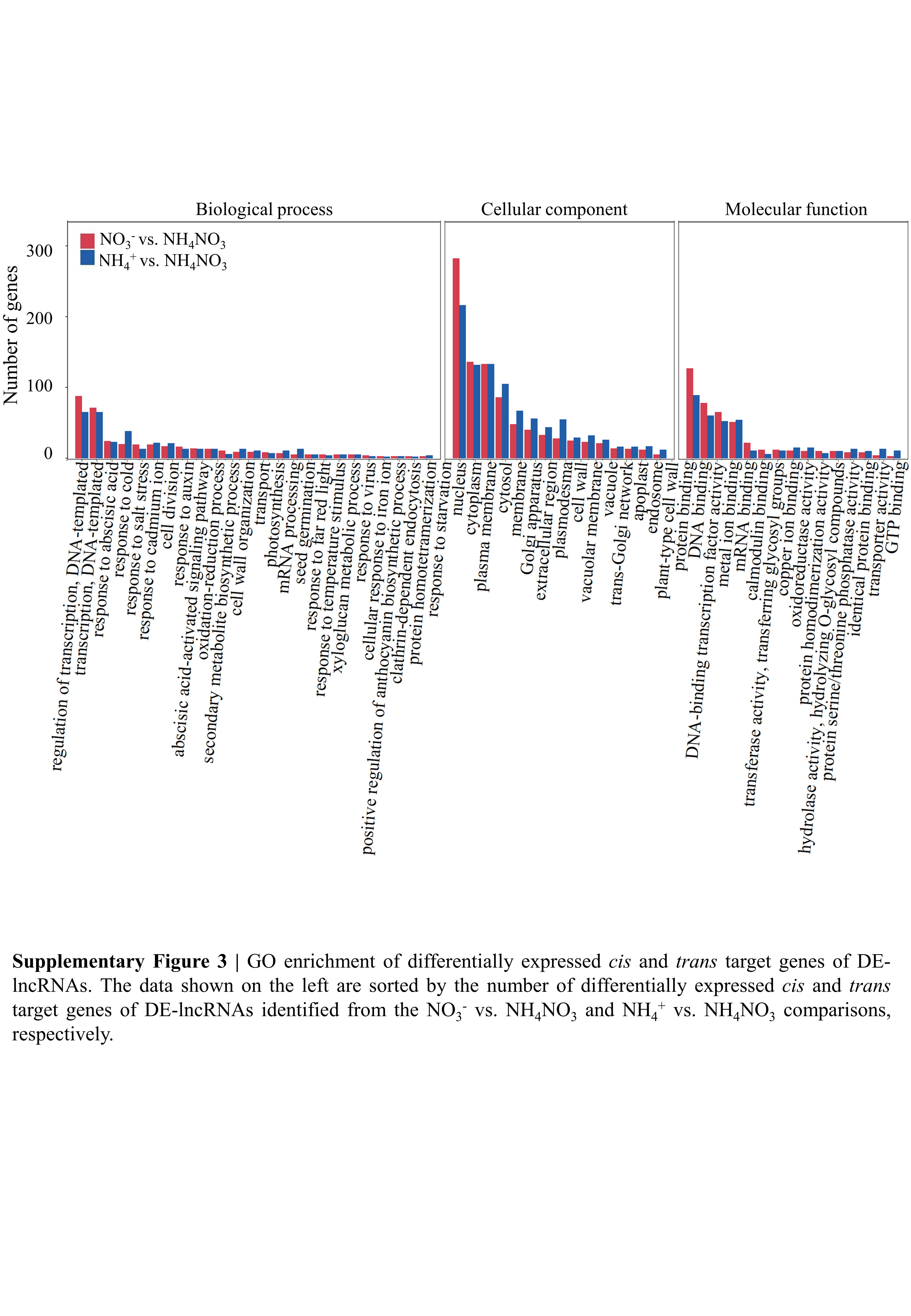

Supplement: Supplementary file 4 [file Image_3.TIF]

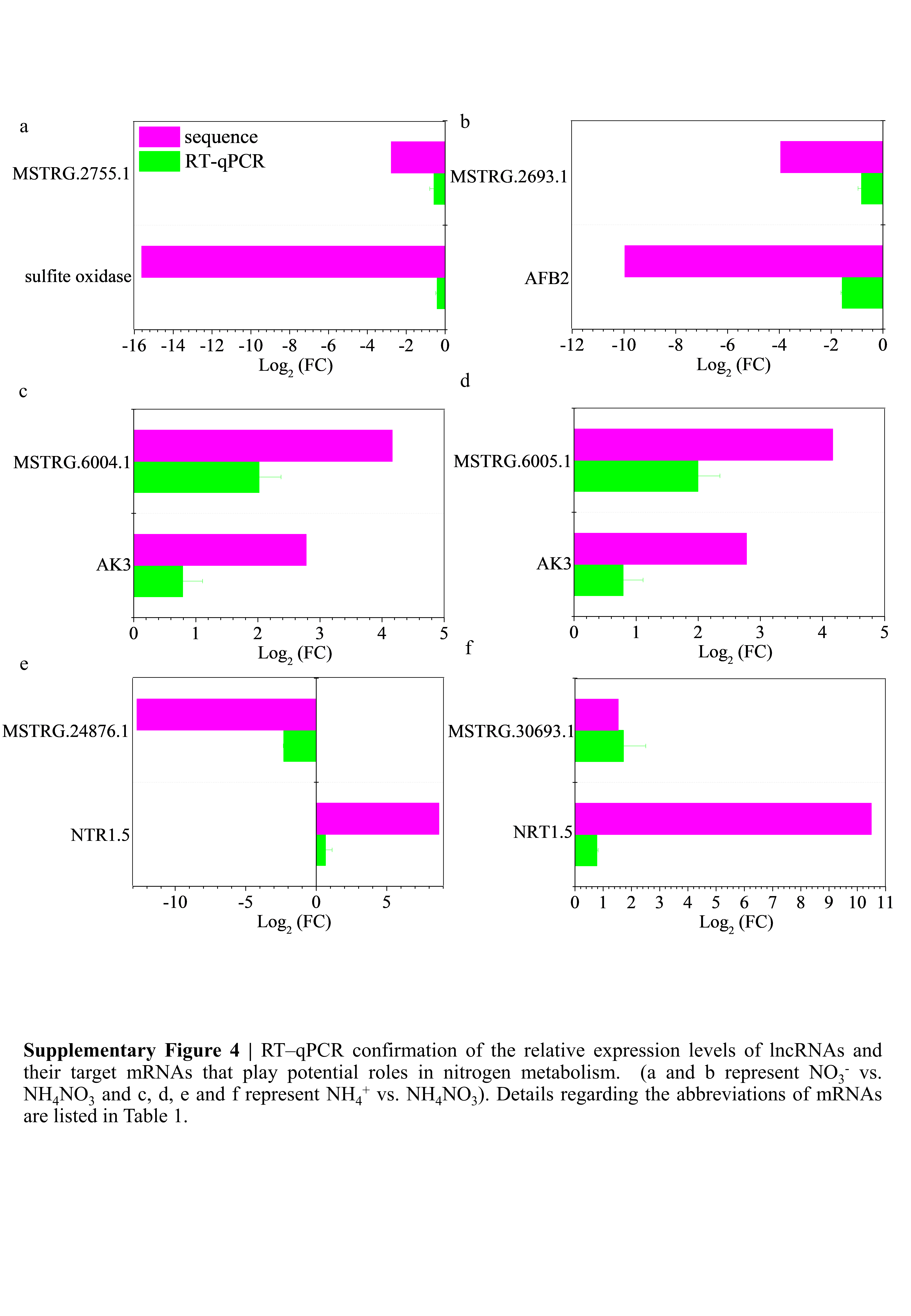

Supplement: Supplementary file 5 [file Image_4.TIF]
